# Supplementary material for: Exploring the Impact of the Linker Length on Heat Transport in Metal–Organic Frameworks
Source: Nanomaterials (Basel). 2022 Jun 22;12(13):2142. doi: 10.3390/nano12132142 (PMC9268455; doi:10.3390/nano12132142)
Supplement: Supplementary file 1 [file nanomaterials-12-02142-s001.zip › nanomaterials-1740672-supplementary.pdf]

Supplementary Materials

Exploring the impact of the linker length on heat transport in metal-organic frameworks

Sandro Wieser [1], Tomas Kamencek [1,2], Rochus Schmid [3], Natalia Bedoya-Martínez [4] and Egbert Zojer [1,*]

[1] Institute of Solid State Physics, Graz University of Technology NAWI Graz, Graz 8010, Austria; sandro.wieser@student.tugraz.at, tomas.kamencek@tugraz.at
[2] Institute of Physical and Theoretical Cemistry, Graz University of Technology, NAWI Graz, Graz 8010, Austria
[3] Computational Materials Chemistry Group, Faculty of Chemistry and Biochemistry, Ruhr-University Bochum, Bochum 44801, Germany; rochus.schmid@rub.de
[4] Materials Center Leoben, Leoben 8700, Austria; olganatalia.bedoya-martinez@mcl.at
* Correspondence: egbert.zojer@tugraz.at

Table of Contents

S1. Details for the ab-initio based reference data 2

S2. Fitting of MOF-FF style classical force fields 3

S1.1 Functional form of the FF 3

S2.1 The FF fitting procedure 5

S2.2 Benchmarking of the FFs 7

S3. Setup and evaluation of the Molecular Dynamics simulations 13

S3.1 Calculating the temperature dependent lattice parameters 13

S3.2 Thermal conductivity with non-equilibrium molecular dynamics simulations 14

S3.3 Finite size effects in heat transport direction 16

S3.4 Finite size effects perpendicular to the heat transport direction 17

S3.5 Evaluation of thermal resistance contributions 18

S3.5.1 Thermal conductivity predictions based on thermal resistance contributions 21

S3.6 Thermal conductivity of IRMOF-1 with the UFF4MOF and the Dreiding FF 22

S4. Analysis of force constants and phonons of the investigated systems 23

S4.1 Comparisons of the force constants in the investigated systems 23

S4.2 Projected densities of states 24

S4.3 Phonon band structures 27

S5. Setup and evaluation of the model systems 30

S5.1 Setup of the model systems 30

S5.2 NEMD simulations 34

S5.3 Projected density of states....................38

S5.4 Parameter scaling....................39

References. 41

This document primarily serves to describe the technical details and processes for producing the values in the main paper. Additionally, some extended analysis is given for certain properties of the investigated systems. To find precise input files for the simulations evaluated here visit the NOMAD Repository and Archive in https://www.doi.org/10.17172/NOMAD/2022.06.02-1.

S1. Details for the ab-initio based reference data

The force fields used in this work were fitted based on reference data obtained from density functional theory (DFT) calculations. These were carried with the Vienna Ab-Initio Simulation Package *VASP* (version 5.4.4)[1–4] employing the following standard pseudopotentials [5,6] for the Perdew-Burke-Ernzerhof (PBE) functional [7] within the projector-augmented wave method. The specific versions of the pseudopotentials used for the individual elements can be seen in Table S1.

Table S1. PAW pseudopotential used for the individual elements for the reference calculations.

Element	PAW Pseudopotential Title
H	PAW_PBE H 15Jun2001
C	PAW_PBE C 08Apr2002
N	PAW_PBE N 08Apr2002
O	PAW_PBE O 08Apr2002
Zn	PAW_PBE Zn 06Sep2000

The occupation of electronic states was described with a Gaussian smearing with a distribution width of $\sigma = 0.05$ eV. The sampling of the reciprocal space and the plane wave energy cutoff were adjusted for each system to converge the total energy below 1 meV per atom. Based on careful and extensive convergence tests, this approach yields highly reliable converged vibrational properties for IRMOFs [8] and organic semiconductors[9]. The system-specific settings can be found in Table S2. It should also be noted that the space group for the simulated unit cells are not consistent. While this is obvious for the tetragonal MOF-1-1-14, also the cubic and otherwise similar IRMOF-10 shows a lower symmetry than the other systems.

Table S2. System-specific cutoff and mesh sampling settings and the space group of the respective used unit cell for the DFT reference calculations.

System	***k*-mesh sampling (-centered)**	Plane Wave Energy Cutoff [eV]	Space group
IRMOF-1	2×2×2 (→ 3 irreducible ***k***-points)	800	Fm-3m
IRMOF-10	1×1×1 (→ 1 irreducible ***k***-point)	800	F432
IRMOF-14	1×1×1 (→ 1 irreducible ***k***-point)	800	Fm-3m
IRMOF-16	1×1×1 (→ 1 irreducible ***k***-point)	800	Fm-3m
MOF-1-1-14	2×2×2 (→ 3 irreducible ***k***-points)	800	I4/mmm

For all systems, an SCF energy convergence criterion of 10^{-8} eV and the global precision parameter *Accurate* (for details see the *VASP* manual)[10] were used (keyword PREC). Moreover, projection operators were evaluated in reciprocal space (*VASP* keyword LREAL set to False), which we found to be crucial to obtain reliable frequencies. Grimme's D3 dispersion correction with Becke-Johnson damping (D3-BJ)[11,12] was employed to treat van der Waals interactions in the systems. As was pointed out by some of us in a detailed study about crystalline naphthalene[9], the PBE functional in combination with the D3-BJ correction results in highly accurate lattice parameters and phonon properties in all frequency ranges especially for molecular crystals.

For fitting atomic charges with the *REPEAT*[13] method as inputs for the force fields (FFs), the local potential (*LOCPOT*) was generated with *VASP*. These local potentials were explicitly chosen to include only electrostatic contributions (ionic and Hartree), omitting exchange and correlation (by setting the variable LVHAR to True).

The *phonopy*[14] code was used to calculate the interatomic force constants and vibrational Γ-phonon modes using primitive unit cells and the default displacement distance of 0.01 Å, which we have usually found suitable for MOFs[8] and organic semiconductors[9]. The harmonic force constants were symmetrized with *phonopy*'s internal routines to account for the acoustic sum rules[15].

S2. Fitting of MOF-FF style classical force fields

The fitting of the force fields (FFs) was performed with the *ff_gen* [16] software, which was also used to obtain MOF-FF [17] force fields in previous works. This section serves to outline the functional form of the FF, the procedure of the fitting and the subsequent benchmarking of the resulting force field potential. All fits were performed on reference data for the primitive unit cell of the respective MOFs. To clarify the used thermology of primitive and conventional cell in this work they are shown in Figure S1 for IRMOF-1. The conventional unit cell is related to the primitive cell via a $\begin{pmatrix} -1 & 1 & 1 \\ 1 & -1 & 1 \\ 1 & 1 & -1 \end{pmatrix}$ supercell matrix.

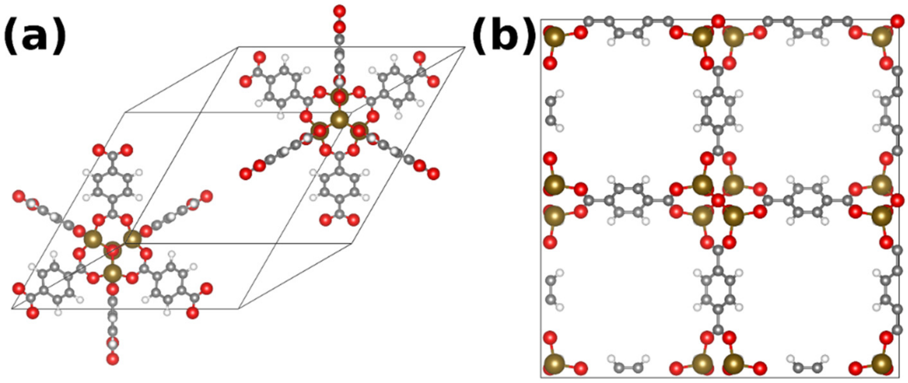

Figure S1. Primitive (a) and conventional (b) unit cell of IRMOF-1. Atomic color coding: Zn…brown, C…grey, H…white, O…red. The linkers in (a) were extended beyond the cell boundaries for clarity. Only 6 linkers are fully contained within the primitive unit cell.

S1.1 Functional form of the FF

The force field is based on mm3 style FFs [18], which were originally designed for organic molecules. It shares many of the bonding FF terms of the MM3 FF. These are divided in bond stretch, angle bends, proper torsional bends and improper torsional bends and have the following functional form for the energy E:

$$E_b^{stretch} = \frac{1}{2}k_b^s\left(r_b - r_b^{ref}\right)^2 \cdot \left[1 - 2.55\left(r_b - r_b^{ref}\right) + \frac{7}{12}\left(2.55\left(r_b - r_b^{ref}\right)\right)^2\right] \quad \text{(S1)}$$

$$E_a^{bend} = \frac{1}{2} k_a^b \left(\theta_a - \theta_a^{ref}\right)^2 \cdot [1 - 0.14\left(\theta_a - \theta_a^{ref}\right) + 5.6 \cdot 10^{-5}\left(\theta_a - \theta_a^{ref}\right)^2$$
$$-7 \cdot 10^{-7}\left(\theta_a - \theta_a^{ref}\right)^3 + 2.2 \cdot 10^{-8}\left(\theta_a - \theta_a^{ref}\right)^4] \quad \text{(S2)}$$

$$E_t^{torsion} = \sum_n \frac{V_t^N}{2} [1 + \cos(n\phi_t + \phi_t^n)] \quad \text{(S3)}$$

$$E_o^{improper} = \frac{1}{2} k_o^i \theta_o^2 \quad \text{(S4)}$$

Here, r_b is the bond distance, θ_a is the angle between three bonded atoms, ϕ_t is the torsion angle, θ_o is the improper torsion angle (out-of-plane bending angle), ϕ_t^n indicates a phase shift for the torsions and k_b^s, k_a^b, V_t and k_o^i are force constants that are to be fitted by *ff_gen*. All variables denoted with *ref* indicate reference values referring to the equilibrium situation. They are usually also fitted with *ff_gen*. The bond stretch and angle bending terms contain fixed anharmonicities which are taken from the original MM3 FF[18].

Additionally, it has been found to be beneficial to include cross terms describing the interactions between two neighboring bonds (str-str), the interaction between bonds and adjacent angles (str-bnd) and the interaction between the next-nearest bonds in a torsional interaction (bb13). The latter represents an extension to the parameters used originally in MOF-FF.

$$E_a^{str-str} = k_a^{ss}\left(r_{a1} - r_{a2}^{ref}\right)\left(r_{a2} - r_{a2}^{ref}\right) \quad \text{(S5)}$$

$$E_a^{str-bnd} = \left(\theta_a - \theta_a^{ref}\right) \cdot \left[k_a^{sb1}\left(r_{a1} - r_{a1}^{ref}\right) + k_a^{sb2}\left(r_{a2} - r_{a2}^{ref}\right)\right] \quad \text{(S6)}$$

$$E_t^{bb13} = N_t\left(r_{ij} - r_1^{ref}\right)\left(r_{kl} - r_3^{ref}\right) \quad \text{(S7)}$$

Here, k_a^{ss}, k_a^{sb1}, k_a^{sb2} and N_t are force constants that need to be fitted. The non-bonded terms for electrostatic and van-der-Waals (vdW) interactions are described in analogy to the MOF-FF[17] in the following way: electrostatic interactions are given by the classical electrostatic potential between two Gaussian charge distributions:

$$E_{ij}^{coul} = \frac{1}{4\pi\epsilon} q_i q_j \frac{\mathrm{erf}\left(\frac{d_{ij}}{\sigma_{ij}}\right)}{d_{ij}} \quad \text{(S8)}$$

Here, d_{ij} is the interatomic distance, q_i and q_j are the atomic charges, σ_{ij} is the Gaussian charge distribution width and ϵ is the dielectric constant. Atomic charges were obtained from the electrostatic potential obtained in the periodic DFT-based reference calculations using the REPEAT method [13]. The charges were averaged over all symmetry-identical atoms in the respective system. The numerical values can be extracted from the available parameter files as part of the Supplementary Materials and as part of the simulation input in the associated NOMAD repository (see above). The values for σ_{ij} were taken from the original MOF-FF [17].

Van-der-Waals interactions (and close-range repulsion) are modeled via a "dispersion-damped" Buckingham potential [17]:

$$E_{ij}^{vdW} = \epsilon_{ij}\left\{1.85 \cdot 10^5 \exp\left(-12\frac{d_{ij}}{d_{ij}^0}\right) - 2.25\left(\frac{d_{ij}^0}{d_{ij}}\right)^6 \left[1 + 6\left(\frac{0.25 d_{ij}^0}{d_{ij}}\right)^{14}\right]^{-1}\right\} \quad \text{(S9)}$$

Here, ϵ_{ij} is the vdW well depth and d_{ij}^0 is the vdW minimum distance. These parameters were taken from the MM3 FF[18].

S2.1 The FF fitting procedure

The fits were performed with ff_gen based on the DFT obtained force constants in redundant internal coordinates (RICs) and the atomic reference coordinates. For the projection of force and position vectors from Cartesian coordinates onto internal coordinates, Wilson's B matrix was used:

$$B_{ij} = \frac{\partial q_i}{\partial x_j} \tag{S10}$$

Where q_i are the internal coordinates and x_j are the Cartesian coordinates. The inverse of the B matrix is then used to convert the Hessian matrix H (= force constant matrix) into internal coordinates:

$$H_{int} = H_{cart} B^{-1} \tag{S11}$$

Which of the terms listed in section S2.1 were used for the individual systems has been adjusted system-specifically depending on the chemical environment (especially for the torsions). For some of the reference bond distances (the metal-oxygen interactions) hard limits were set to prevent unphysical parameters. The other parameters were left unconstrained. The fits were performed for at least 5000 generations (depending on convergence speed more generations were required) of the covariance matrix adaptation evolution strategy (CMA-ES[19]) fit with a population size of 64.

The objective function Z that is minimized by the algorithm arises from a reference-FF mean square deviation sum over various parameters (str = bond stretches, ibe = in plane bending angles, obe = out-of-plane bending angles, tor = torsions, hes = Hessian):

$$Z = Z_{str} + Z_{ibe} + Z_{obe} + Z_{tor} + Z_{hes} + Z_{lattice} \tag{S12}$$

Where the individual elements with the exception of $Z_{lattice}$ take the form

$$Z_p(FF) = \frac{w_p}{M_p} \sum_{i=1}^{N_p} \omega_i \left(r_i(FF) - r_i^{ref} \right)^2 \tag{S13}$$

Here, w_p is the weight of the interaction type or the entire Hessian, ω_i are separate weights for the individual values, M_p is the sum over all ω_i and r_i represent the individual values in internal coordinates (this can be distances, angles or force constants). For the case of the Hessian, the total contribution to Z is further split up in individual contributions corresponding to the internal coordinate type (str, ibe, obe and tor). For each of these types, individual weights can be assigned if needed. Additionally, cross terms, which describe interactions between different internal coordinates are only considered in the fitting process if an appropriate force field term is added to the potential definition. This applies, for example, to the str-str cross terms describing interactions between two bonds. The remaining non-diagonal elements of the Hessian are not directly fitted, but many of them are close to zero and the comparisons provided in the next section in Figure S3 shows their agreement in addition to the directly fitted terms. The values of the Hessian also influenced the types of potential terms used in the force field. Some FF definitions, like COMPASS[20], also include additional cross-terms for four atom groups between torsion angles, bending angles and bond distances. However, all of the corresponding elements in the Hessian matrix were zero, which is why only the bb13 terms were included for the systems investigated in this work. The cross terms are not bound to a specific weight for the fit but their weight depends on the type of internal coordinate they are associated with. The str-str and str-bnd terms have the same weights as the ibe terms and the bb13 terms the same weight as the tor terms. However, the total weight of an ibe or tor term is normalized by the number of involved interactions. Since the force field is intended to be fitted to data for a well relaxed structure at 0 K, it makes sense to also consider the lattice parameters during the fit. However, properly optimizing the lattice parameters for every

FF sample is computationally very expensive. This is why the lattice has been considered using the stress tensor S using the following form:

$$Z_{lattice} = w_{stress}\left(\frac{1}{9}\sum\left(C^{-1}\cdot(S\cdot V)\right)^2\right)^2 \quad (S14)$$

Here, V is the cell volume, C is the cell tensor and w_{stress} is once again a weight. It is clear that the total objective function is made up of different quantities with different dimensions which will all impact Z differently. This is why it is important to assign the weights such that all criteria contribute to a similar order of magnitude. The weights for the individual parameters were all chosen to be equivalent, but differences still arise between the individual interaction types due to a varying number of parameters. Table S3 shows the chosen weights for the interaction types in the fits in addition to the total contribution to the objective function at the end of the fit for IRMOF-1. It can be seen that despite the small weight assigned to the lattice stress contribution, the value still comes as close to zero as the recorded digits. The largest contribution toward the objective function is given by the Hessian contribution, which has been assigned a higher weight as an accurate description of the Hessian is crucial to model phonons. In order to improve the typically poorly fitted Hessian components, higher weights were used for obe and tor.

Table S3. Weights chosen for the FF fit for the contribution towards the total objective function Z. The weighted final contributions at the end of the fit are also given for IRMOF-1

Z contribution type	weight	final Z contribution for IRMOF-1
bond stretches	1.0	0.00000001
in-plane bending angles	1.0	0.00000001
out-of-plane bending angles	1.0	0.00000000
torsion angles	1.0	0.00000001
Hessian in relevant RICs	5.0	0.00000133
➢ str Hessian contributions	1.0	0.00000003
➢ ibe Hessian contributions	1.0	0.00000016
➢ obe Hessian contributions	10.0	0.00000004
➢ tor Hessian contributions	10.0	0.00000013
➢ cross Hessian contributions	-	0.00000097
lattice stress	0.001	0.00000000

Another relevant setting for the fit is given by the starting values and especially starting intervals used for the fit parameters. These are used in the CMA-ES implementation, where a multivariate normal distribution function is applied over the previous (or at the start of the fit, the initial) value. This distribution depends on a global deviation parameter σ that is dynamically modified by the fitting algorithm and on the variation range set for each parameter. That means that if the initial variation ranges are distributed unevenly across the individual variables, a situation could emerge that some variables fluctuate heavily over the course of the fit while others remain close to constant. This behavior can sometimes also be exploited to encourage certain parameters like bond lengths to remain within physically reasonable intervals. The inclusion of a hard limit for specific variables serves as an additional tool to control the fit. This is mostly used to make sure that all fitted non cross-terms are positive but also applied to some particularly problematic parameters to prevent unphysical FFs. However, the choices made here are not entirely consistent for all systems as these settings primarily arose from trial and error in an iterative fitting process. One common source of problems, especially when trying to fit cross terms, is a proper description of the reference bond lengths. Using a small starting range for these parameters around the equilibrium bond length from the DFT reference is very helpful to reach a physically reasonable FF. It can also be useful to only fit the cross terms once the reference bond lengths and angles were fixed based on the results of a previous fit (an approach that was used for IRMOF-14).

Another setting that proved to be very useful for all the systems was to add a penalty for modes of very low frequency. For this a multiplicative factor of 1000 was added to the objective function Z if the lowest frequency optical mode was below a certain threshold. For our systems these thresholds depended on the lowest frequency at Γ in the DFT reference. For each system they were: IRMOF-1: 19 cm^{-1}, IRMOF-10: 10 cm^{-1}, IRMOF-14: 5 cm^{-1}, IRMOF-16: 6 cm^{-1} and MOF-1-1-14: 10 cm^{-1}. This additional penalty was added to make sure that the fit properly converges to a realistic minimum and does not get stuck at intermediate solutions that include imaginary frequencies.

For the special case of IRMOF-10 there was consistently unsatisfactory agreement of the FF to random DFT reference forces using the same methodology. It appears that the fit tended to diverge towards a poor minimum that included unphysical parameters. To remedy this, the differences of the forces for 100 random displacements computed with DFT and the forces obtained by the FF were included in the objective function for the fit. The displacements were generated by applying a Gaussian displacement distribution over each Cartesian coordinate component of each atom in the relaxed system. The standard distribution of the Gaussian ranged from 0.01 Å to 0.1 Å in steps of 0.01 Å with 10 different structures for each distribution width. This additional reference data add an additional contribution to the objective function Z:

$$Z_{forcematch} = \frac{1}{N} w_{forcematch} \sum_n \omega_n \sum_i \left(F_{ir} - F_{ir,0}\right)^2 . \quad \text{(S15)}$$

Here, n is the index over the individual reference structures, N the total number of these structures, i is the index of the internal coordinates, ω_n is a weight that can be set for each individual structure and F_{ni} are the forces in internal coordinates. The weight $w_{forcematch}$ was set to 10^{-7} and each of the reference systems contributed equally to $Z_{forcematch}$. Including force matching led to a similar agreement of the forces to reference data as the other systems, which is most likely related to a reduced probability of drifting into a minimum with some unphysical parameters.

However, the most severe problems during the creation of a force field arise from a suboptimal definition of the interatomic potential. If one uses a reliable (but slow) algorithm like CMA-ES a reasonable agreement with the reference data can typically be achieved for as long as degrees of freedom exist that can affect the target quantities. For further details regarding the benchmarking showing the quality of the fits, see the next section.

The resulting parameters can be found in the associated Nomad Repository and as part of the Supplementary Materials.

S2.2 Benchmarking of the FFs

This section serves to show the agreement of the FFs to DFT reference data. The primary reference point will be geometrical parameters, forces, and phonon-related properties.

In Figure S2 the agreement of the bond lengths, reference angles, torsion angles and out-of-plane bending angles is shown. In Figure S3 the agreement of the Hessian matrix (= force constant matrix) in internal coordinates is shown for the investigated systems. Table S4 lists the root mean square errors for these properties for the investigated systems. Figure S4 shows the agreement of the phonon frequencies at the G point for the investigated systems.

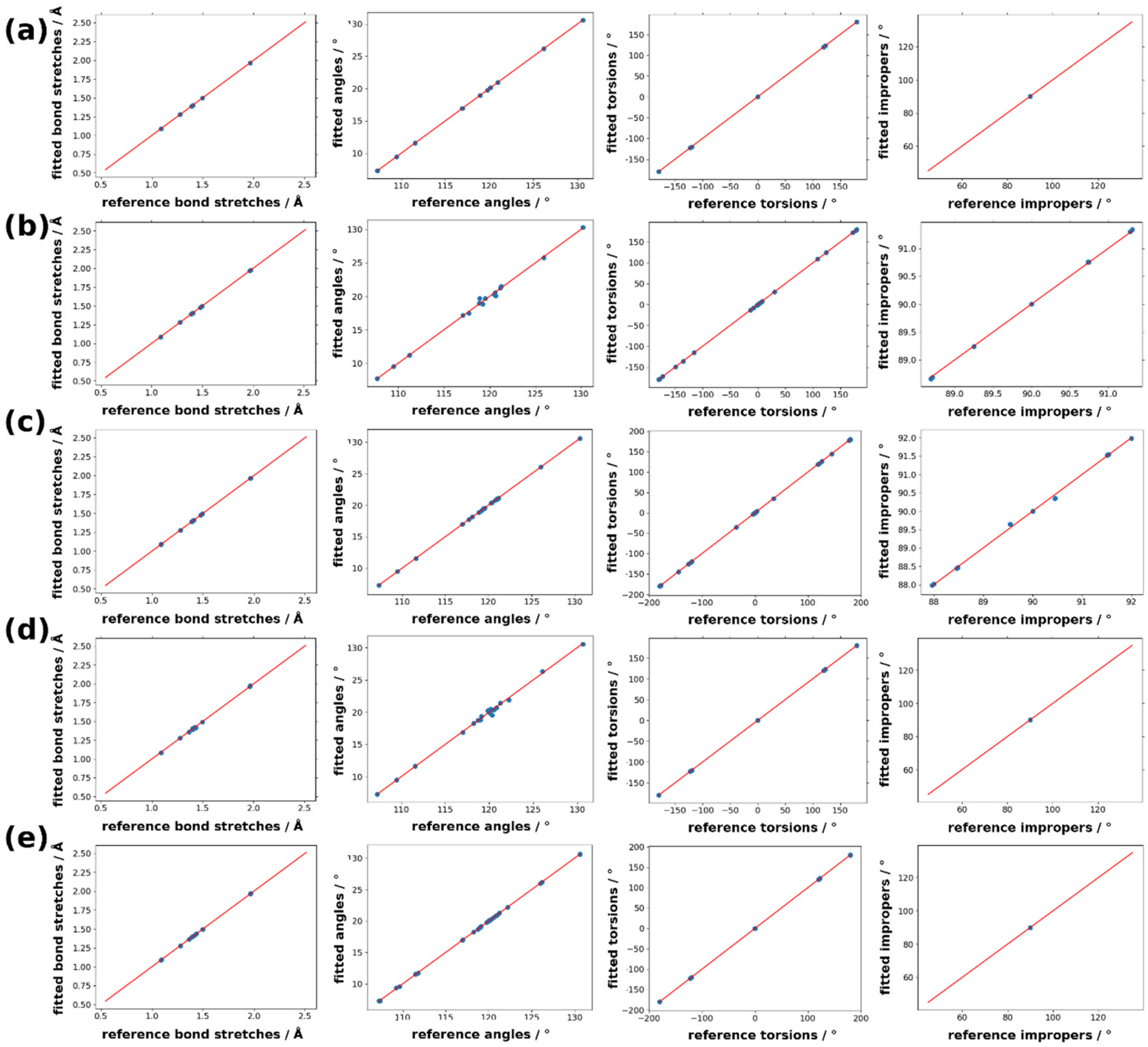

Figure S2. Comparisons of bond lengths, bending angles, torsion angles, and improper torsion angles between the fitted FF and the DFT reference for (a) IRMOF-1, (b) IRMOF-10, (c) IRMOF-16, (d) IRMOF-14 and (e) MOF-1-1-14.

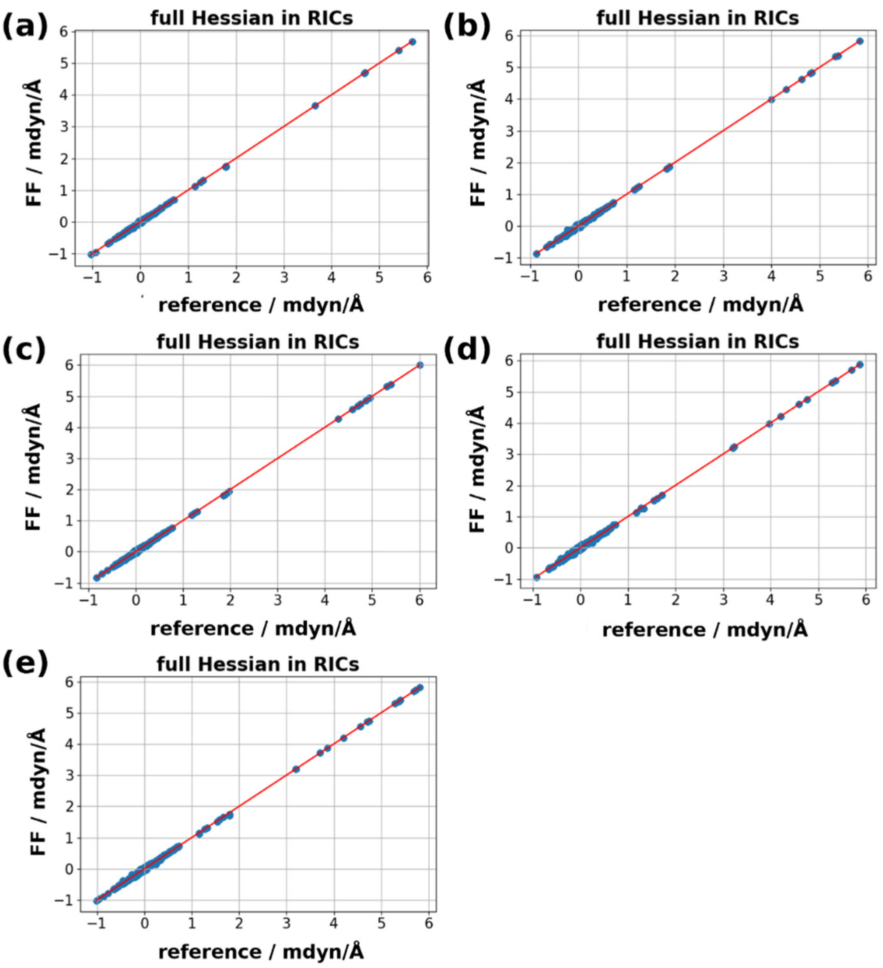

Figure S3. Comparisons of the Hessian matrix in redundant internal coordinates (RICs) between the fitted FF and the DFT reference for (a) IRMOF-1, (b) IRMOF-10, (c) IRMOF-16, (d) IRMOF-14 and (e) MOF-1-1-14.

Table S4. Root mean square deviations of properties in the force field compared to the DFT reference.

Property	IRMOF-1	IRMOF-10	IRMOF-16	IRMOF-14	MOF-1-1-14
**bond lengths / Å**	0.0001	0.002	0.00007	0.009	0.0006
**angles / °**	0.006	0.3	0.003	0.2	0.04
**proper torsions / °**	0.005	0.03	0.04	0.007	0.07
**improper torsions / °**	0.001	0.02	0.05	0.000002	0.0007
**hessian / kcal Å^{-2} mol^{-1}**	0.003	0.002	0.002	0.002	0.002
**frequencies / cm^{-1}**	13.9	16.7	25.2	15.4	12.5

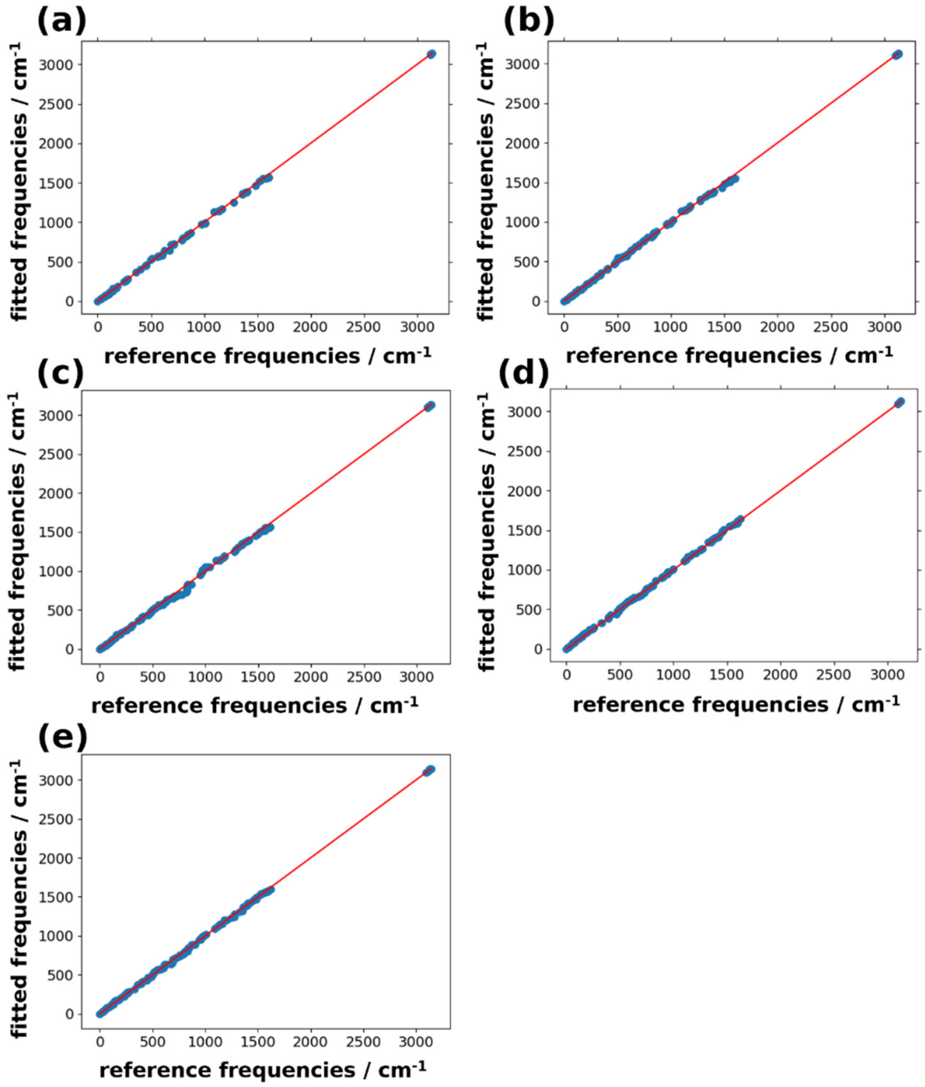

Figure S4. Comparisons of the Vibrational frequencies at the G-point between calculations employing the fitted FF and the DFT reference for (a) IRMOF-1, (b) IRMOF-10, (c) IRMOF-16, (d) IRMOF-14 and (e) MOF-1-1-14.

In Figure S5, histograms of the dot products between the phonon eigenvectors at G from the DFT reference data and those obtained from the fitted FFs are shown. Many occurrences of eigenvector overlaps close to 1.0 indicate a high level of similarity of the modes computed by the different approaches. The G-modes of the FFs and of the DFT reference data were assigned relying on the approach described in Ref. [9].

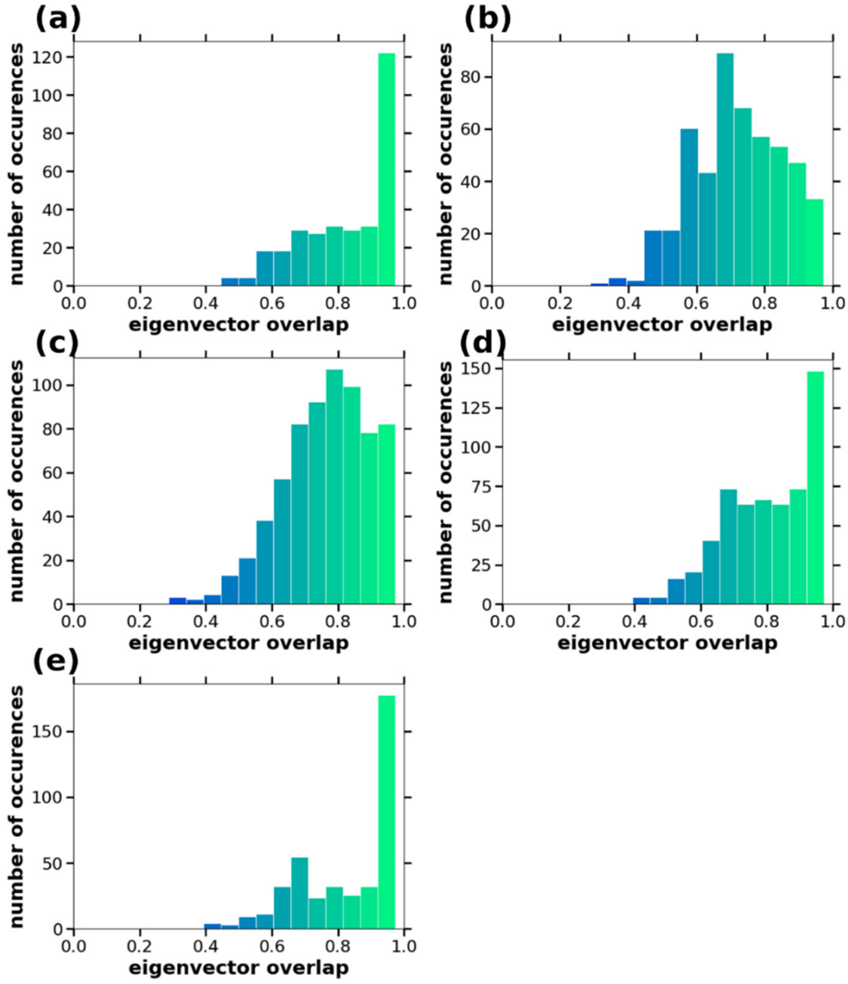

Figure S5. Histograms of the dot products of the best fitting eigenvectors between the fitted FF and the DFT reference for (a) IRMOF-1, (b) IRMOF-10, (c) IRMOF-16, (d) IRMOF-14 and (e) MOF-1-1-14.

Figure S6 visualizes a comparison between the forces for random displacements of atoms calculated with the FF and the DFT-based reference forces. They have been calculated from 30 randomly displaced structures. Since for the case of IRMOF-10, these forces were actually used to obtain the FF (see above), for this system displacements along the eigenmodes are also included (see Figure S7). The reason for that is that solely considering forces that were previously used already in the FF fit would give the wrong impression of exceptionally small errors in such a plot. Finally, Figure S8 visualizes the phonon band structure for IRMOF-1 obtained with the FF and compares it to DFT-based reference data by Rimmer et al. from [21]. It should be noted, that in [21], no dispersion correction was applied, which is in contrast to our simulation, where the Grimme D3-BJ correction has been used (see section S1). While a proper treatment of van der Waals interactions is not as important for IRMOF-1 as, for example, for organic crystals, the neglect of van der Waals interactions by Rimmer et al. could still be responsible for some of the deviations (albeit the impact is presumably rather small – see also [8]).

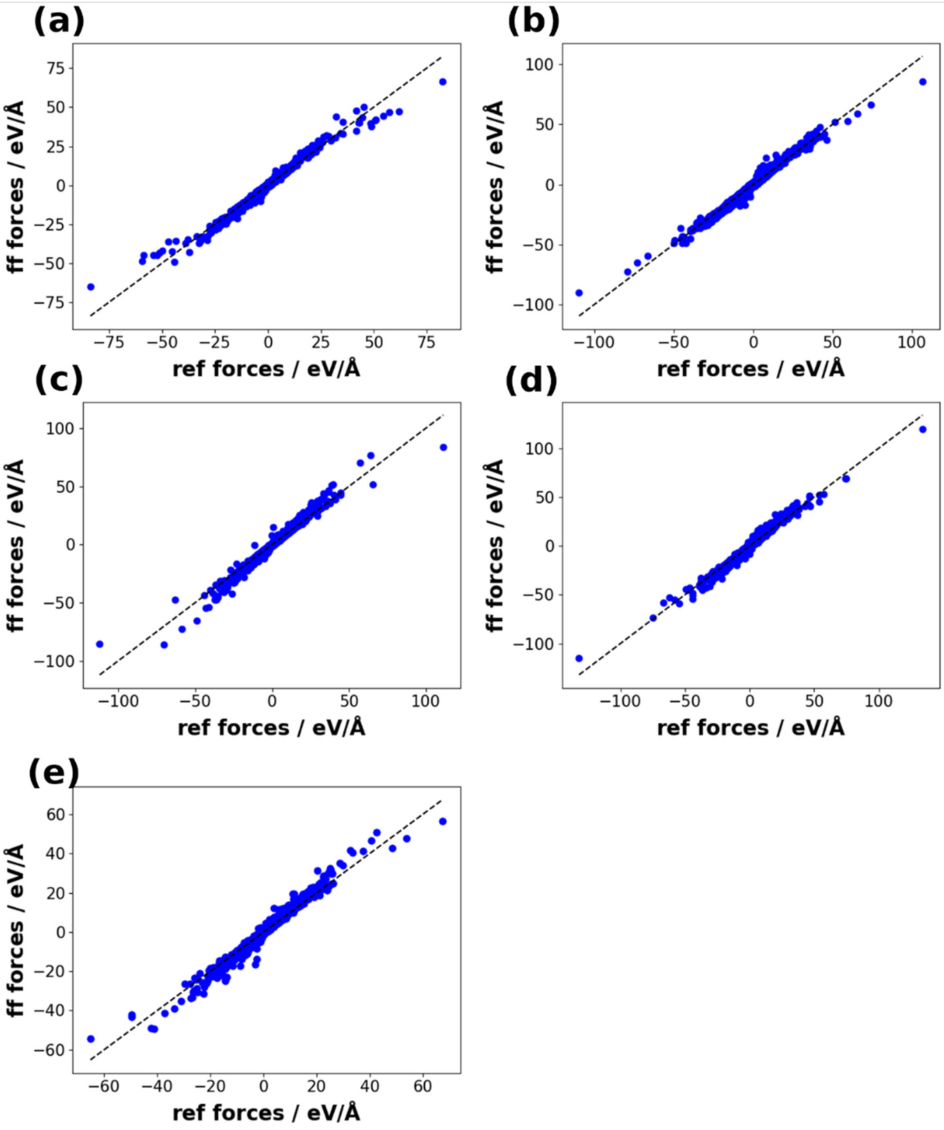

Figure S6. Comparisons of forces on all atoms for random displacement structures calculated with the fitted FF and the DFT reference methodology for (a) IRMOF-1, (b) IRMOF-10, (c) IRMOF-16, (d) IRMOF-14 and (e) MOF-1-1-14.

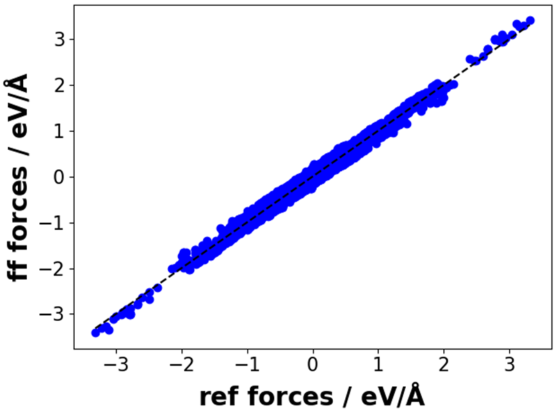

Figure S7. Comparison of forces on all atoms for displacements along the low frequency eigenvectors calculated with the fitted FF and the DFT reference methodology for IRMOF-10.

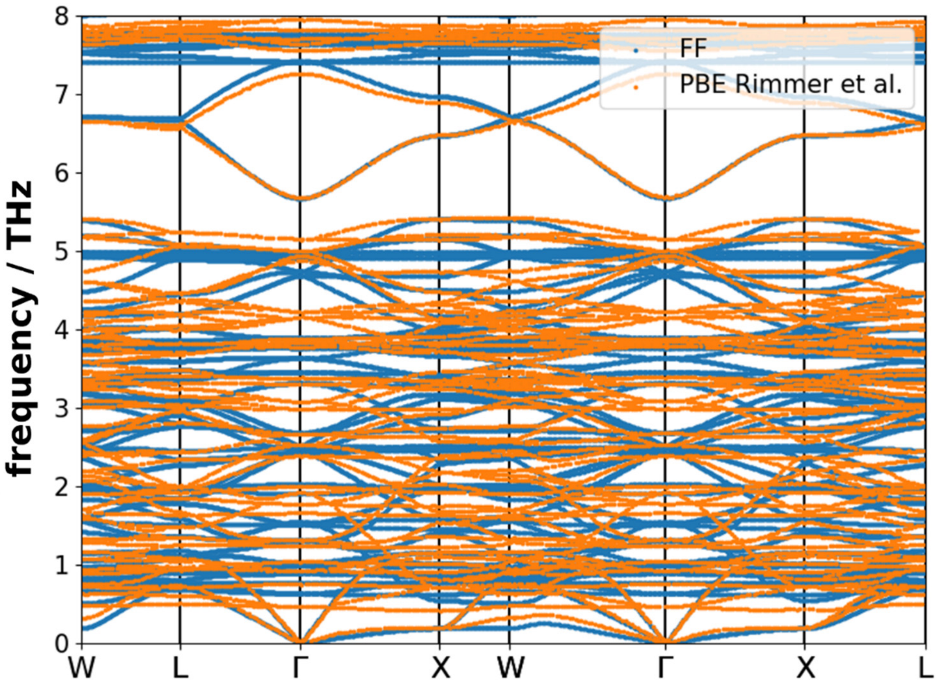

Figure S8. Comparison of the phonon band structure for IRMOF-1 obtained with the fitted FF and with Density functional perturbation theory by Rimmer et al.[21].

S3. Setup and evaluation of the Molecular Dynamics simulations

This section describes the procedure and convergence of the molecular dynamics (MD) simulations. In general, contributions from the Buckingham and electrostatic potential were only considered up to a global cutoff of 12 Å. A smoothing for both of the potentials was invoked within 90% of the cutoff. Long range interactions were considered for all molecular dynamics simulations for the real MOFs using a particle-particle particle-mesh (pppm) solver [22] with a precision of $5 \cdot 10^{-6}$ kcal/mol. Since the model systems do not include any long ranging non-bonded interaction terms, the pppm solver was not applied for these systems.

S3.1 Calculating the temperature dependent lattice parameters

In order to account for temperature dependent thermal expansion, MD simulations in the isobaric-isothermic (NPT) ensemble were performed to obtain the lattice parameters at the working temperature of 300 K. For this, 2×2×2 cells of the conventional cubic unit cell were used and the lattice parameters, which were allowed to change anisotropically, were recorded every 100 time steps (with a time step of 0.5 fs) after a brief equilibration phase of 50 ps. The simulations were run for 2.5 ns. The lattice parameters were averaged over the remaining simulation time after the equilibration phase and over symmetry-equivalent directions. The resulting values for the lattice parameters for the (conjugate gradient energy- and forces- relaxed) cell at 0 K and for the NPT simulations at 300 K are shown in Table S5. The table also shows the thermal expansion for the systems which were obtained from a linear fit over several NPT simulations for temperatures ranging from 100 K to 400 K. This is reasonable, because in this temperature range the temperature dependence of the lattice parameters is very close to being linear, as can be seen in experiments for IRMOF-1 [23] and in our simulations in Figure S9.

Table S5: FF-optimized lattice parameters at 0 K (via conjugate gradient optimization) and 300 K (via NPT molecular dynamics), a_{0K} and a_{300K}, respectively; additionally values for average linear thermal expansion, a, in a temperature range between 100 and 400 K are listed for the investigated systems. They were obtained based on average lattice parameters from molecular dynamics simulations in an NPT ensemble.

System	a_{0K} / Å	a_{300K} / Å	α / 10^{-6}K^{-1}
IRMOF-1	26.07	25.95	-15.67
IRMOF-10	34.79	34.69	-11.56
IRMOF-14	34.58	34.49	-9.10
IRMOF-16	43.35	42.98	-28.19
MOF-1-1-14	34.59	68.83	-18.10
MOF-1-14-1	26.07	51.91	-16.42

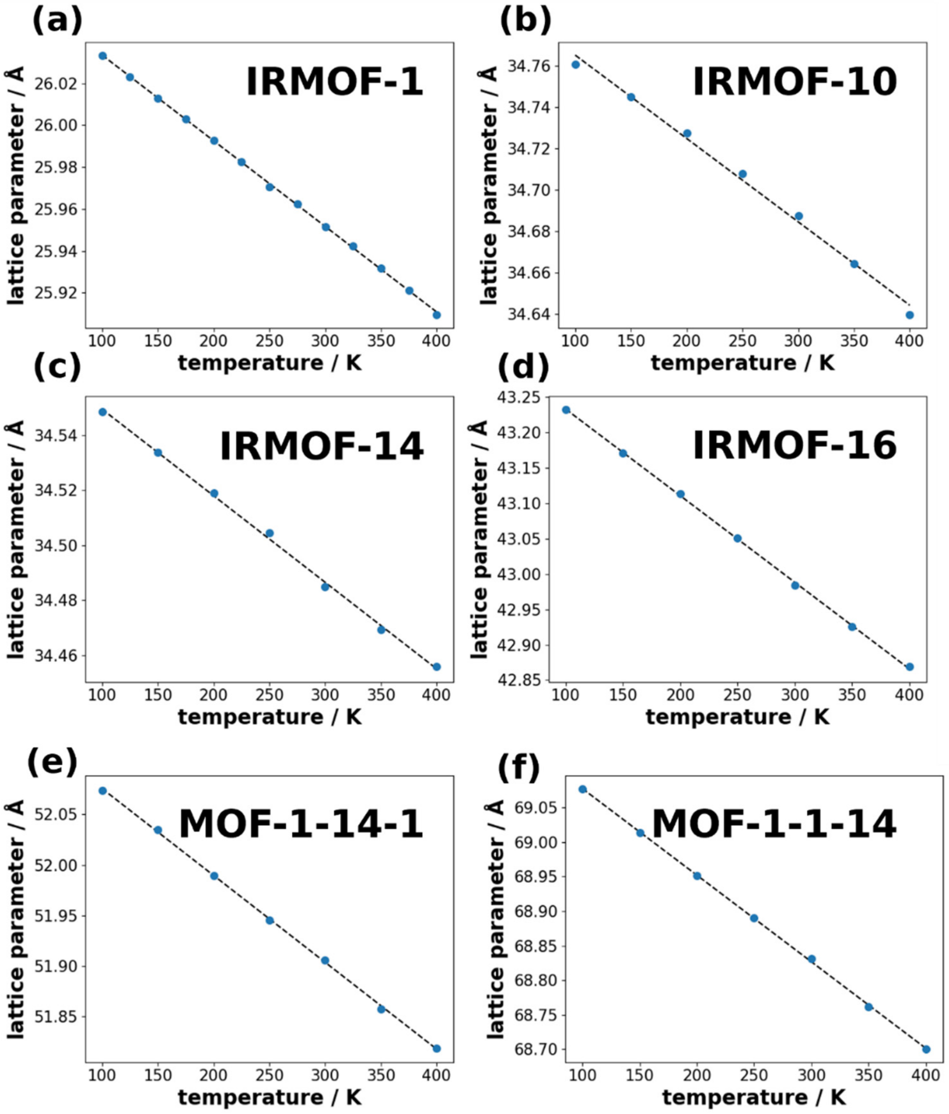

Figure S9. Temperature dependent lattice parameters of the conventional unit cell obtained from NPT molecular dynamics simulations for a 2×2×2 supercell of the following systems: (a) IRMOF-1, (b) IRMOF-10, (c) IRMOF-14, (d) IRMOF-16, and (e, f) MOF-1-1-14. For the latter, (e) shows the short lattice parameter (BDC direction) and (f) shows the long lattice parameter (pyrene direction). The dashed lines indicate the linear fits to obtain the thermal expansion coefficients.

S3.2 Thermal conductivity with non-equilibrium molecular dynamics simulations

The non-equilibrium molecular dynamics (NEMD) simulations were performed at 300 K using supercells of the systems with the lattice parameters that were obtained for

this temperature. First, an (isothermal-isochoric) NVT simulation was performed using a Langevin thermostat with a damping parameter of 100 fs to raise the system temperature to 300 K for at least 250 ps. Over this duration the average total energy of the system is computed. Additional time steps were computed until the relative deviation of the total energy of the most recent time step from the previously obtained average energy was smaller than 10^{-8}. This last time step close to the average energy represented the starting point for the actual NEMD simulation. This served to more accurately reach the energy needed to maintain an average temperature of 300 K and noticeably reduced the statistical error of the NEMD simulations. If such an energy matching process was not done, one would carry out the further simulation at a larger or lower temperature depending on the current state of the energy fluctuation.

Subsequently, two Langevin thermostats were applied at the center and the edges of the systems (see Figure 2 of the main manuscript). The thickness of the thermostats always amounted to half of a conventional cubic (or tetragonal for the case of MOF-1-1-14) unit cell of the respective MOF or one cubic unit cell of a model system. This was chosen to realize consistent positions of thermostat barriers for all the systems. The bulk of the system is described in an NVE ensemble and the simulations were performed for 20 million time steps of 0.5 fs (amounting to 10 ns in total). During this time, the kinetic energies, the local temperatures and the energy added and subtracted by the thermostats were recorded. These values were then averaged over time after reaching a steady state. To ensure the latter, the data from the initial ~10 % of the simulation time was discarded. The heat flux J was directly computed by the energy added/subtracted by the thermostats E_{therm} and the area of the cross section of the simulation cell A with

$$J = \frac{1}{2}\frac{E_{therm}}{A}\ . \tag{S16}$$

The factor 2 arises from the fact that heat flows in two directions from the central, hot thermostat to the two cold thermostats in the periphery (see Figure 2 in the main manuscript). The temperature profiles were computed from the time averaged kinetic energies of an atom using

$$\overline{T'} = \frac{3}{2k_B}E_{kin}. \tag{S17}$$

The temperature gradient to obtain the thermal conductivity was obtained by the temperature difference given by the Langevin thermostats as shown in Figure S10 and the distance between the thermostat boundaries. This is different from the more conventional approach to just fit the linear temperature gradient in the bulk of the system (indicated as a black line). Considering the whole region between the thermostats has been proposed by Li et al. who showed that the temperature jump at the thermostat boundary should not be ignored to properly model the thermal conductivity, as long as local thermostat methods are used in the simulation[24]. Local thermostat methods in this context refers to approaches in which atomic velocities are adjusted stochastically and locally and not as a global rescale parameter applied to all atoms in the thermostat region; the Langevin thermostat has been proven to perform well for NEMD simulations [24].

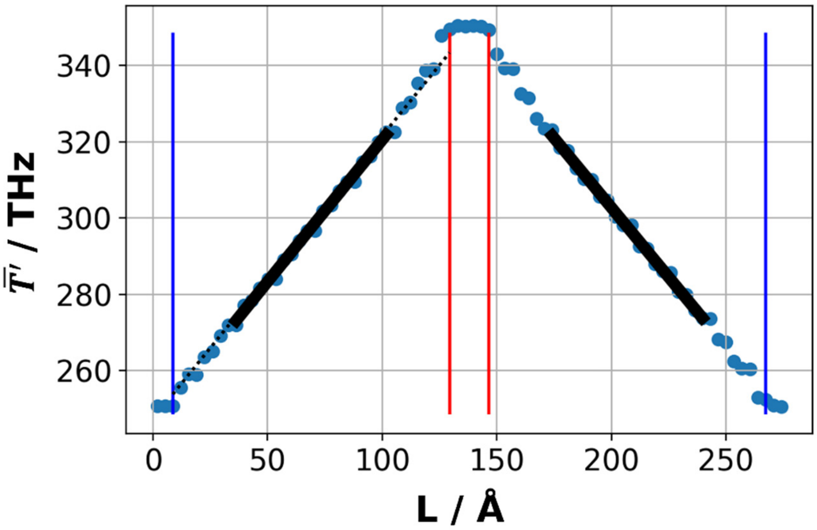

Figure S10. Averaged temperature profile during a NEMD simulation of an 8x2x2 conventional supercell of IRMOF-14. The red vertical lines indicate the location of the hot thermostat boundaries and the blue vertical lines the location of the cold thermostat boundaries. The black lines indicate the location and slope of the linear fit traditionally used to obtain the temperature gradient. However, we instead use the temperature difference between the hot and the cold thermostat and the distance between the thermostat boundaries for calculating the temperature gradient, following the suggestion by Le et al.[24].

A concern that can be raised when performing NEMD simulations is the large temperature difference of 100 K between the hot and cold thermostats leading to unrealistically large temperature gradients. Additionally, the thermal conductivity is generally temperature dependent and larger temperature differences can impact the results. Conversely, a large temperature gradient is beneficial for analyzing the local effective temperature profiles due to a reduced influence of noise from thermal fluctuations. The temperature dependence of IRMOF-1 has already been shown both experimentally and computationally to be very low which should dispel these concerns [25,26]. Still, to also ensure that we obtain reliable values for the thermal conductivities, NEMD simulations were also carried out for temperature differences ΔT of 50 K and 150 K. The resulting thermal conductivities for an 8×2×2 supercell of IRMOF-1 were 0.191 W/mK and 0.193 W/mK respectively compared to 0.191 W/mK for a ΔT of 100 K.

S3.3 Finite size effects in heat transport direction

The non-equilibrium molecular dynamics simulations were performed for several cell lengths in order to account for finite size effects. For each of the systems at least 5 different cell sizes were used, but for some systems more were added to reduce the statistical error. The thermal conductivity values for each simulation were evaluated and their inverse values $1/\kappa$ as a function of the inverse length $1/L$ are shown in Figure S11 for all systems. The slope in these plots should be linear and after performing a linear fit through the data points, the intercept at $\frac{1}{L} = 0$ can be interpreted as the thermal conductivity in the infinite size limit[27]. The error bars in the main text result from the standard error of this intercept value arising from the fit.

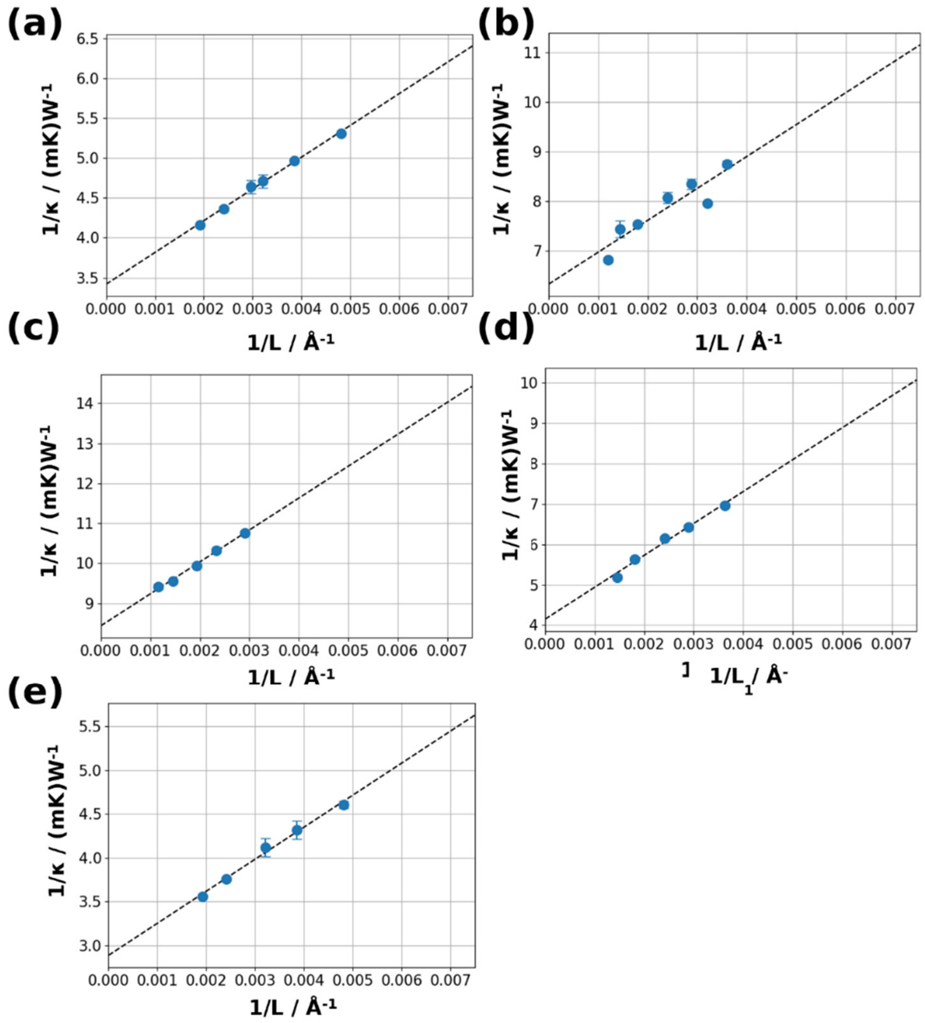

Figure S11. Inverse thermal conductivities as a function of inverse cell lengths (blue) obtained from NEMD simulations for the investigated systems: (a) IRMOF-1, (b) IRMOF-10, (c) IRMOF-16, (d) IRMOF-14 and (e) MOF-1-1-14. The dashed lines indicate the linear fit through the datapoints. The finite size corrected thermal conductivities can be extracted from the 1/L=0 intercept.

S3.4 Finite size effects perpendicular to the heat transport direction

Additionally, it is crucial to explore the impact of the cell diameter in the cross-section perpendicular to heat-flux direction. A very small cell diameter can lead to a significantly increased thermal conductivity compared to the actual value. Tests have been performed for IRMOF-1 and IRMOF-14 regarding the impact of the cell diameter on the thermal conductivity and it was found that 1×1 cells are insufficient to accurately describe the thermal conductivity, but that the difference between 2×2 and 3×3 cells is relatively minor in comparison: for IRMOF-1 the finite size corrected thermal conductivities were (0.41 ± 0.02) W/(mK), (0.293 ± 0.008) W/(mK) and (0.290 ± 0.008) W/(mK) for 1×1, 2×2 and 3×3 super cells respectively and for IRMOF-14 (0.24 ± 0.02) W/(mK) and (0.23 ± 0.02) W/(mK) for 2×2 and 3×3 super cells in the cross-sectional direction. As can be seen, the differences between the 2×2 and 3×3 cells are within the statistical error of the infinite size extrapolation.

It should be noted that in our previous work about thermal transport in IRMOFs [28], a coincidence during the convergence tests led us to conclude that 1×1 cells are already sufficient. While we did test NEMD simulations for different cell diameters perpendicular to heat transport direction, this was only done for one cell length in heat transport direction. However, the slope of the fit to the infinite size limit also depends on the supercell size perpendicular to the heat transport direction. So, despite a very similar value for the thermal conductivity for 8×1×1 and 8×2×2 cells, we now found that the final finite size corrected thermal conductivity for the 8×1×1 is actually not fully converged. This is why

in [28], we somewhat overestimated the absolute values of the thermal conductivities compared to the real values. For example, for IRMOF-1 we reported a thermal conductivity value of 0.41 W/(mK) which is substantially higher than the properly converged value of 0.29 W/(mK) obtained in this work. In this context it should be noted that one can show that, since in [28] only rather similar systems have been compared, this does not impact the trends discussed there.

S3.5 Evaluation of thermal resistance contributions

The thermal resistance contributions were obtained from the local effective temperature profile calculated from the atomic kinetic energies. The time-averaged kinetic energies were additionally averaged for equivalent atoms with the same distance from the thermostat region (i.e. equivalent species in small slabs perpendicular to the direction of the heat flux). This led to an "atomistic effective temperature profile" which was used in the visual representations in this work to identify the positions of highly heat conductive regions (the node and linker). The main barrier for heat transport (largest step in the temperature profile) was identified to be between the metal atoms of the node and the oxygen atoms facing the linker. The position of this barrier was defined to be precisely halfway between these atoms. The temperature gradients required to obtain the thermal resistance contributions for node and linker were computed via a linear fit over all time-averaged local effective temperatures of all atoms in the section enclosed by the previously identified heat transport barriers. The interface resistance was obtained by the remaining temperature difference that were not included in the temperature gradient of adjacent node/linker segments. The local effective temperatures for all occurrences of equivalent segments (node/linker/interface) which are not within a distance of 50 Å from the thermostat centers were used for the fit leading to the final values. The reason for skipping the regions closest to the thermostats boundaries is that there it is not possible to properly evaluate thermal resistance contribution. The local effective temperature profiles of all linker-interface-node-interface-linker "blocks" were centered around their average position and their average temperature. Blocks that are located on one of the sides of the central hot thermostat were mirrored around the zero position, so that all of the blocks can be combined to a single dataset representing the temperature profile of a thermal repeat unit. The fits were then performed over those combined temperature profiles, which are shown in Figure S12. To be consistent with the recipe for the evaluation of the thermal conductivity for a NEMD simulation as proposed by Li et al. [24], where the full temperature profile including the step at the thermostat boundary is considered, a further correction was introduced. As in the evaluation of the thermal resistance contributions the regions closest to the thermostats were not considered, the thermal resistance contributions were linearly scaled by a relative temperature factor $\frac{\Delta T_{full}}{\Delta T_{bulk}}$ representing the difference between the bulk and full temperature difference. ΔT_{bulk} was obtained from the difference between temperatures obtained at the thermostat boundaries when considering linear fits of the position-dependent temperatures in the bulk region away from the thermostats. Conversely, ΔT_{full} is the actual temperature difference between the thermostats. This led to the final thermal resistance values for one NEMD simulation. However, to compare the individual systems with each other, also finite size effects needed to be considered. The correction was performed analogously to the total thermal conductivity first, the effective segment thermal conductivity was obtained from equation 4 in the main manuscript (the effective thermal conductivity results from the length of the repeat unit Δz_{unit} divided by the thermal insulance $r_{A,i}$). This quantity was evaluated as a function of the inverse length of the simulation box in heat transport direction $1/L$ and a linear fit was performed through the computed datapoints. The effective segment thermal conductivity in the infinite size limit was obtained from the $\frac{1}{L} = 0$ intercept. From these, the individual (transport-channel) thermal insulance contributions could be calculated, again, using equation 4 in the main manuscript. The error bars given in the main manuscript result from the standard error of the said fits. Figure S13 shows the individual thermal resistance fits for the three contributions in all investigated systems.

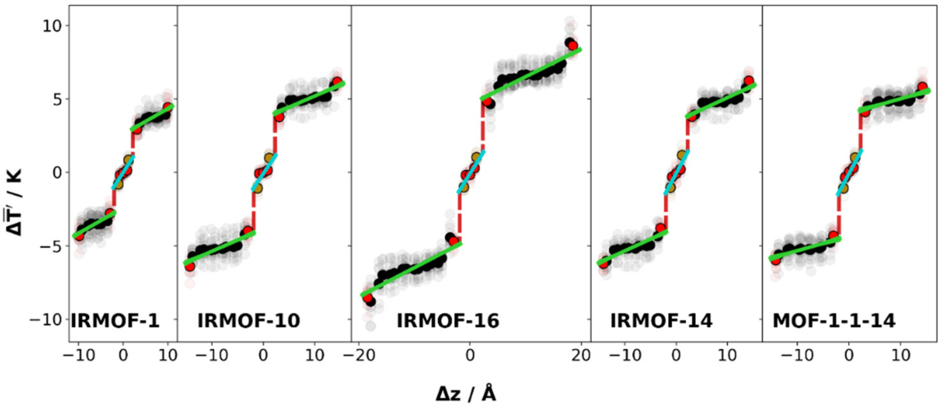

Figure S12. Local effective temperature profiles of a node and its adjacent linkers obtained from NEMD simulations on the investigated MOFs. The temperature profiles were averaged across all MOF building blocks that are at a distance of at least 50 Å from the thermostat center. The transparent data points in the background represent the local effective temperatures before averaging. The total lengths of the simulation cells were chosen to be similar, resulting in the following super cells, specified relative to the conventional unit cells: 13×2×2 for IRMOF-1, 10×2×2 for IRMOF-10, IRMOF-14, and MOF-1-1-14, and 8×2×2 for IRMOF-16. The linear fits to obtain the thermal resistance contributions are shown in green for the linker and in cyan for the node. The interface thermal insulance contribution is obtained from the remaining temperature difference between node and linker, which is indicated in red.

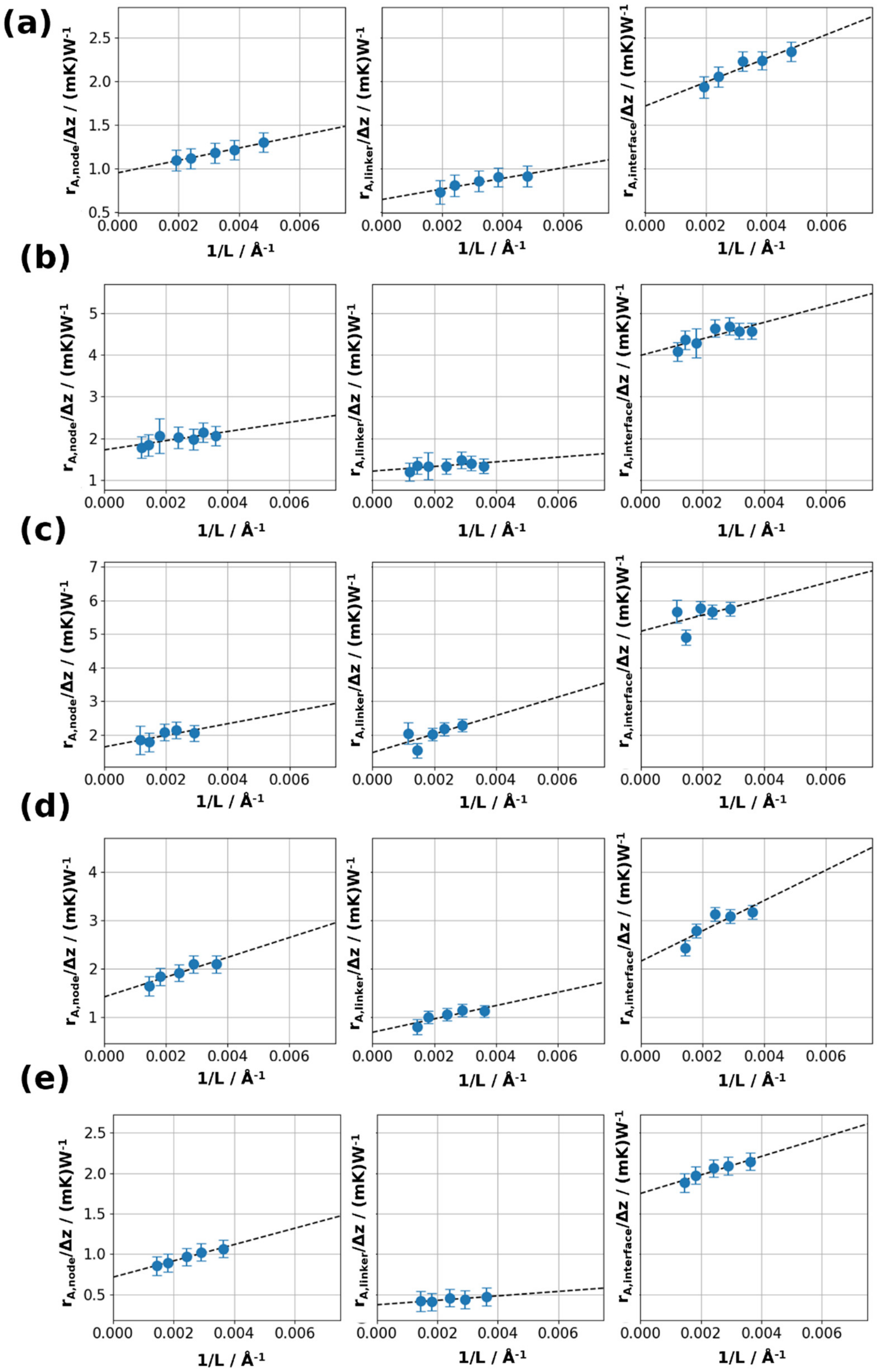

Figure S13. Inverse effective segment thermal conductivities obtained from the thermal resistance contributions for node, linker, and interface divided by the length of the thermal repeat unit Δz as a function of inverse lengths of the simulation box (blue) from NEMD simulations for the investigated systems: (a) IRMOF-1, (b) IRMOF-10, (c) IRMOF-16, (d) IRMOF-14 and (e) MOF-1-1-14. The dashed lines indicate the linear fit through the datapoints. The finite size corrected effective thermal conductivities can be extracted from the 1/L=0 intercept.

S3.5.1 Thermal conductivity predictions based on thermal resistance contributions

In Figures 4 and 6b in the main manuscript, there are also several predictions for the thermal conductivities that are based on the thermal resistance contributions for linker, node and interface. This brief section serves to explicitly provide the equations that were used for the predictions in figures 4 and 6. They are based on equation (7) in the main manuscript and the simple assumption that a reduction of the density of heat transport channels leads to a reduction of the thermal conductivity. The latter is simply modeled by a linear rescaling of the base thermal conductivity κ_0 with the inverse cross-sectional area A_{cross}.

$$\kappa_{pore} = \kappa_0 \cdot \frac{A_{cross,0}}{A_{cross}} \tag{S18}$$

For the predictions based on the thermal resistance contributions, the following expression is used:

$$\kappa_{extend_z} = \frac{\Delta z_{unit} N}{A_{cross}\left(\frac{\Delta z_{linker}}{\Delta z_{linker,0}} r_{N,linker} + r_{N,node} + 2 \cdot r_{N,interface}\right)} \tag{S19}$$

Here, it is assumed that the interface and node transport-channel insulances remain constant and that the total contribution of the linker insulance scales linearly with the linker length Δz_{linker}. The only difference between the models of extending the linker length in all or only in heat transport direction is given by a different cross-sectional area.

When analyzing thermal resistance contributions, it is important that the total thermal conductivity calculated from infinite-size limit thermal resistance contributions matches the total thermal conductivities as obtained directly from the NEMD simulations (i.e., without the separation into individual contributions from nodes, linkers, and interfaces). This comparison is shown in Figure S14 for the investigated systems. It can be clearly seen that the direct NEMD values (black symbols) agree well with the values obtained from the thermal resistance contributions (colored symbols). Residual deviations originate from the statistical errors occurring in the evaluations of the different quantities.

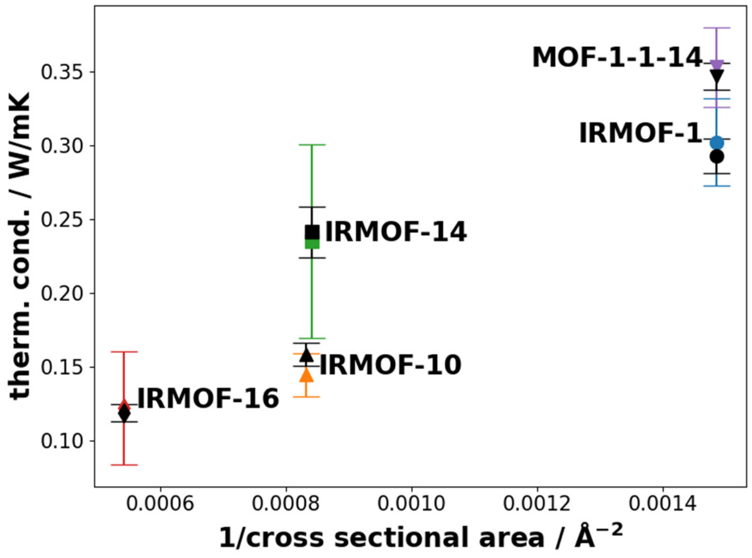

Figure S14. Comparison of the total thermal conductivities obtained from the thermal resistance contributions (colored symbols) and the total thermal conductivities calculated employing Fourier's law (black symbols) as a function of the inverse cross-sectional area of the conventional unit cell of the respective system.

S3.6 Thermal conductivity of IRMOF-1 with the UFF4MOF and the Dreiding FF

In order to show that the quality of force fields is crucial for determining physical properties in materials, we computed the thermal conductivity of IRMOF-1 with two system-unspecific force fields, designed to describe the entire periodic table of elements: the extended universal force field for MOFs (UFF4MOF)[29,30] and the Dreiding force field[31]. The parameters were assigned using the *lammps-interface* tool written by Boyd et al. for MOFs, who also published an extensive comparison of force fields predicting various physical properties for MOFs[32]. The simulation settings used for the molecular dynamics simulations were the same as for the MOF-FF. The lattice parameter at 300 K for IRMOF-1 amounted to 26.186 Å for Dreiding and to 26.388 Å for UFF4MOF. Figure S15 shows the length dependent inverse thermal conductivities for the force fields leading to the finite size corrected thermal conductivity values given in the main paper. Figure S16 shows the phonon band structures for MOF-FF/Dreiding/UFF4MOF explaining the much higher thermal conductivity values for the system-unspecific force fields due to much higher group velocities.

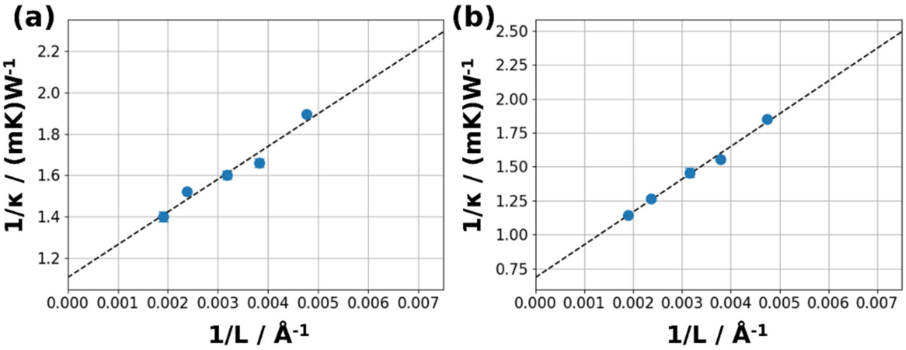

Figure S15. Inverse thermal conductivities as a function of inverse cell lengths (blue) from NEMD simulations on IRMOF-1 with (a) the Dreiding and (b) the UFF4MOF force field potentials. The dashed lines indicate the linear fit through the datapoints. The finite size corrected thermal conductivities can be extracted from the 1/L=0 intercept. The simulations were carried out for 8×2×2, 10×2×2, 12×2×2, 16×2×2 and 20×2×2 super cells of the conventional unit cell of IRMOF-1.

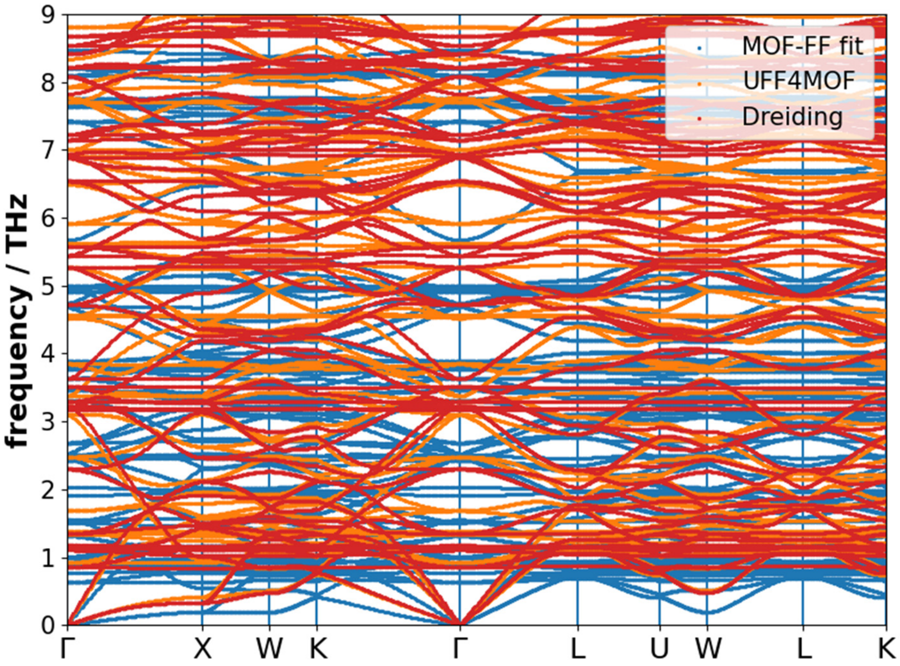

Figure S16. Phonon band structures of IRMOF-1 in the low frequency region calculated employing the following force fields: MOF-FF (blue), UFF4MOF (orange) and Dreiding (red). The phonon band structures were evaluated for a 2×2×2 supercell.

S4. Analysis of force constants and phonons of the investigated systems

In order to compute the force constants and the phonon properties of the investigated systems, the *phonopy* software package was used[14]. For all the systems, a 2×2×2 supercell with a displacement distance of 0.01 Å led to the converged phonon band structure (i.e., to a band structure displaying no visible differences from a band structure calculated for a 3×3×3 supercell). The cell parameters used were those obtained by a 0 K relaxation. In order to evaluate the force constants for specific bonds, they were converted into internal coordinates, as explained in section S2.2.

S4.1 Comparisons of the force constants in the investigated systems

The objective of this section is to present a comparison of force constants in internal coordinates for the bonds certain neighboring atoms. This serves to provide a qualitative overview about the differences in interaction strengths for the individual linker configurations in the MOFs. Figure S17 shows the force constants in internal coordinates for selected bonds between the Zn, O and C atoms for the investigated systems as a function of the lattice parameter of the conventional unit cell. The values are taken from the DFT reference calculations for the primitive unit cell of the respective systems. Due to the good quality of the force field fits (see the comparisons of Hessian matrices in Figure S3), the values reported here are almost identical to the ones that can be computed for the classical force fields. For the interaction between metal and the oxygen from the carboxylate linker (Zn-O(CO2)), the trend of the force constants is not entirely consistent but systems with longer linkers tend to lead to a lower value. Noticeable is, that IRMOF-10 lies clearly below all the other systems indicating that the deviation from the standard node geometry (see Figure 5c in the main paper) leads to a decrease of the interaction strength between node and linker. The force constants for the bond between the metal and the central oxygen atom in the node in Figure S17b does not really seem to show any noteworthy trends and the values are much closer to each other for the different systems compared to the other interactions shown here. For both the bond between the carbon and oxygen atoms in the carboxylate and the bond between the carbon atom of the carboxylate and the nearest carbon atom of the organic linker, the force constants show an almost linear increase with

the cell length. The tetragonal MOF-1-1-14 shows the strongest deviations from the general trend indicating that the different linkers in different directions also directly affect each other's interaction strengths. It should be noted that especially the interactions in the organic part of the MOFs show much higher force constants than the ones including the node atoms. This is not only due to weaker interactions between Zn and O but also strongly related to the higher degree of coordination of the atoms in the node as this trend is also observed in the simple model systems.

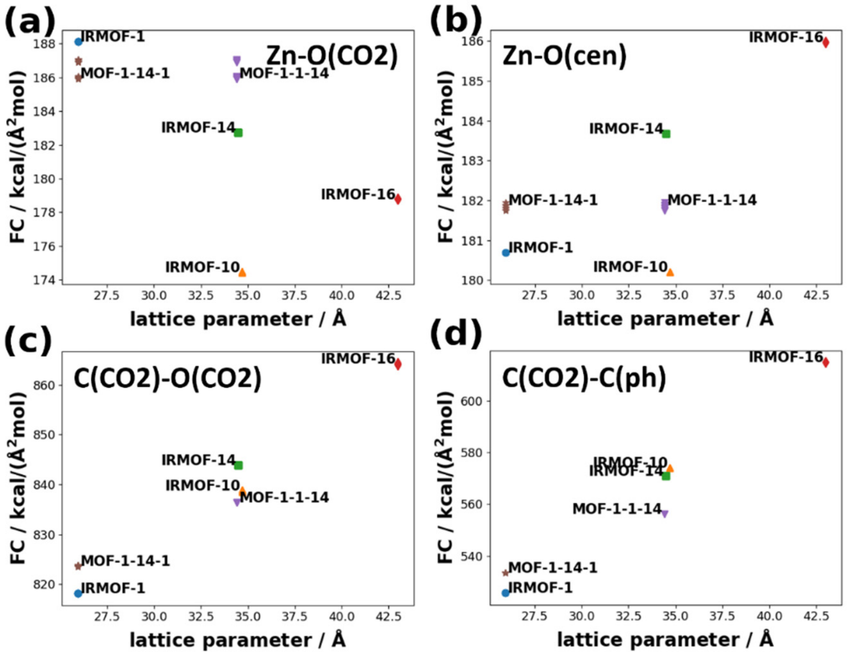

Figure S17. Force constants for the bond stretches of certain bonds in the investigated MOFs with respect to linker length. The shown bond stretches are: (a) the bond between Zn and the O in the carboxylate (Zn-O(CO2)), (b) the bond between Zn and the central O in the node (Zn-O(cen)), (c) the bond between the O and C atoms within the carboxylate (C(CO2)-O(CO2)) and (d) the bond between one C atom from the carboxylate and one the closest C from the aromatic part of the linker (C(CO2)-C(ph)). The values here are taken from the DFT reference force constants.

S4.2 Projected densities of states

To investigate the origin of the heat transport bottleneck at the interface further it can be useful to analyze the phonons in the systems. Phononic heat transport bottlenecks at interfaces between pristine materials can result from a difference in the lattice vibrations of the two connected materials leading to increased scattering. Phonon interface scattering is reduced, when the phonons of both materials are very similar. As an indicator for the similarity of phonon modes in two materials, the phonon density of states are frequently investigated [33,34]. The larger the overlap in the densities of states, the closer matched are the phonons of the individual materials leading to reduced scattering. In our case, we analyze an interface within the unit cell of a crystalline material. The density of states is not directly accessible as a material property in this case. This is why we instead investigated the projected density of states (PDOS) for the node, the linker and the carboxylate group, which is shown for all investigated systems in Figure S18. The PDOSs were computed on a per-atom basis from the eigenvectors evaluated on a 10×10×10 mesh grid of wave vectors (q-points) according to the definition:

$$\mathrm{PDOS}_j = \frac{1}{N}\sum_k \sum_\lambda \delta(\omega - \omega_\lambda)\left|\boldsymbol{k}\cdot\boldsymbol{e}_\lambda^j\right|^2 \tag{S20}$$

Here, N denotes the number of q-points, ω the frequencies, $\boldsymbol{k}$ the Cartesian direction projection vectors **x**, **y** and **z**, and $\boldsymbol{e}$ are the eigenvectors for atom j describing the mode λ for. Since the density of states is only evaluated on a discrete mesh, the delta distribution is approximated by a Gaussian distribution with a standard deviation (smearing parameter) of 0.1 THz. The per-atom projected densities of states were then summed over the groups of atoms associated with certain MOF components, like the central parts of the linkers (phenylene or pyrenylene), the node (zn4o) or the carboxylates (co2) bonding the linkers to the nodes. Please note that the following discussion will also contain some qualitative insights obtained from observing the vibrations at Γ in an animated fashion. The supplementary materials also contain anime.ascii files for each system storing the information regarding the displacements in real space associated to each phonon mode at Γ that can be visualized using a software called v_sim.

The densities of states of the different MOFs in Figure S18 show some clear similarities: for all systems one finds a significant low frequency peak, strong contributions from the carboxylates in the 2-4 THz regions, and a relatively small consistent contribution by the nodes for all low frequency modes with larger peaks at 4-6 THz. However, it becomes clear that all components of the MOFs contribute towards the DOS in this frequency region. Nevertheless, some trends can still be discerned. The PDOSs for the longer linkers generally shift to lower frequencies and for IRMOF-10 the lowest linker-related peak splits into two separate peaks. This is connected to a reduced symmetry of this system. Additional peaks emerge at higher frequencies for systems with an increased number of phenylene rings at 2 THz for IRMOF-10 and at 1.5 THz and at 2.4 THz for IRMOF-16. These are dominated by an out-of-phase twisting of the phenylenes relative to each other. The pyrene linker for IRMOF-14 and for MOF-1-1-14 does not show additional prominent peaks in the low frequency region due absence of easily rotatable linker segments. Note, that all these peaks primarily emerge from relatively localized rotations of the linkers. Thus, these modes show a relatively low dispersion and are unlikely to significant contribute to heat conduction. Still, they can interfere with other highly dispersing modes of similar frequency and lead to a reduction in thermal conductivity.

The zn4o and co2 components do not really show very clear trends upon changing the linker length. The Zn_4O nodes are responsible for a very prominent peak for IRMOF-1, that broadens for the systems with different linkers. The peaks associated with the carboxylate connector appear to shift in frequency for the different system. A clear identification of carboxylate-related DOS peaks is, however, difficult, as almost all vibrations in the considered frequency range involve motions of the carboxylate, including the additional modes added by the more complex linkers.

In the end, we can only really observe clear trends for one of the components in the interface, the organic linker. When trying to derive some conclusions regarding the overlap of the PDOSs a qualitative comparison becomes a lot more difficult. This is why we compute an overlap similarity index I in the same way as in [35]:

$$I_{ij} = \frac{\int_0^{\omega_{max}} PDOS_i(\omega) PDOS_j(\omega) d\omega}{\sqrt{\int_0^{\omega_{max}} PDOS_i(\omega)^2 d\omega \int_0^{\omega_{max}} PDOS_j(\omega)^2 d\omega}}. \quad \text{(S21)}$$

Where ω_{max} is the maximum frequency until which the overlap index should be evaluated and i and j stand for the different densities of states that should be compared. In our case, we limited the comparison to the overlap between node and linker (co2 + ph or pyren). The values for the overlap similarity index are shown as a function of the thermal conductivity and as a function of the interface transport-channel insulance in Figure S19 including the low frequency modes up to 9 and up to 3 THz respectively. For the frequency region up to the first 9 THz no discernable trends can be seen for the overlap index as a function of both, the thermal conductivity, and the interface transport-channel insulance. When only investigating the very low frequency modes with frequencies up to 3 THz (which should be more relevant for heat transport), one can discern a consistent trend excluding the data point for MOF-1-1-14. For the latter system only the PDOS of the

pyrene based linker with the node was used in the evaluation of the overlap index, which can explain a deviation from the trend due to a reduced number of considered linkers. However, even excluding MOF-1-1-14, we observe a reduction of the overlap index with an increase in the thermal conductivity and an increase of the overlap index with the interface transport-channel insulance. This trend is the exact opposite of what one might have expected. It is also somewhat problematic that the rather arbitrary choice of the maximum frequency to compute the overlap index changes the trends completely. However, these findings are not that surprising when considering that we are dealing with very complex systems with many different phonon modes – many of which barely carry any contributions toward the thermal conductivity due to their low group velocities (see section S4.3). Still, all these modes significantly contribute to the DOS. This is why it is difficult to draw conclusions based on the densities of states alone. One would have to carry out an in depth analysis for individual relevant phonon modes by computing their individual contributions toward the thermal conductivity, which exceeds the scope of this work.

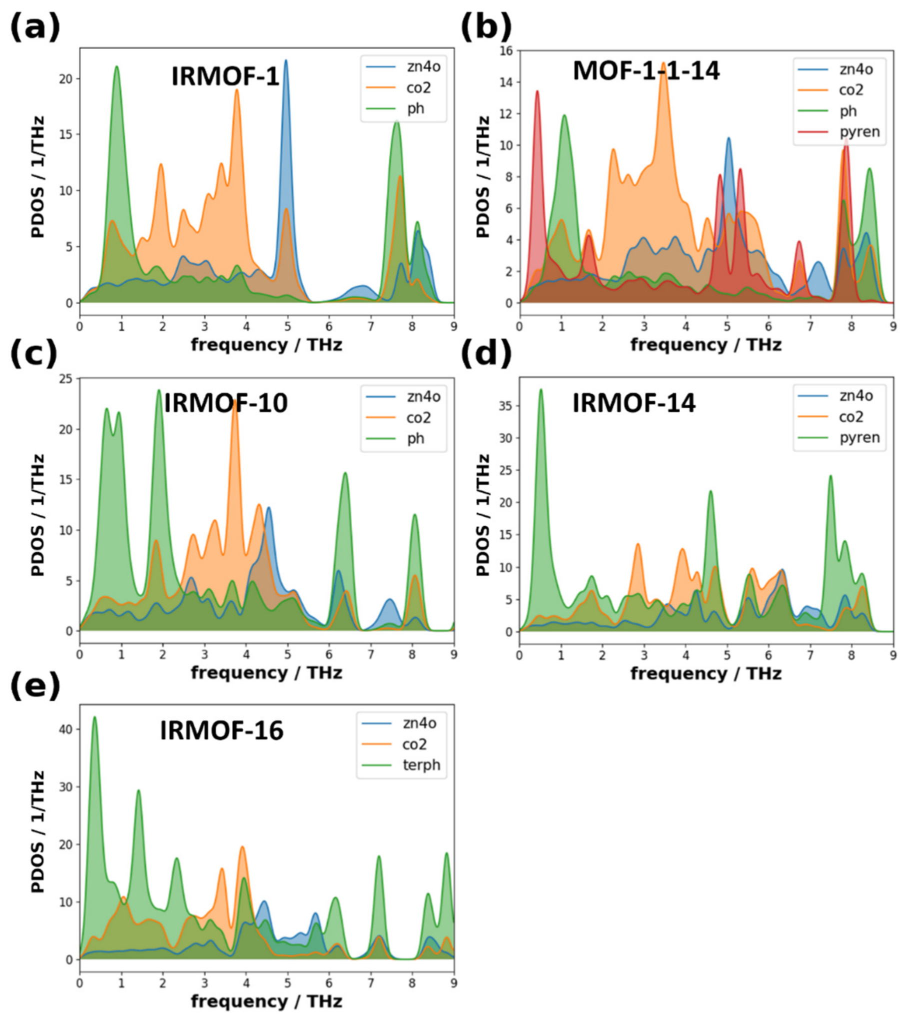

Figure S18. Contributions of different components of the MOFs to the phonon density of states for the investigated systems: (a) IRMOF-1, (b) MOF-1-1-14, (c) IRMOF-10, (d) IRMOF-14 and (e) IRMOF-16. The considered components are the node (zn4o), the carboxylate group (co2) and the rest of the linkers (ph, terph or pyren). The data were obtained using a 10x10x10 mesh of q-points with frequencies calculated using *phonopy*[14] employing forces derived from the MOF-FF type force field.

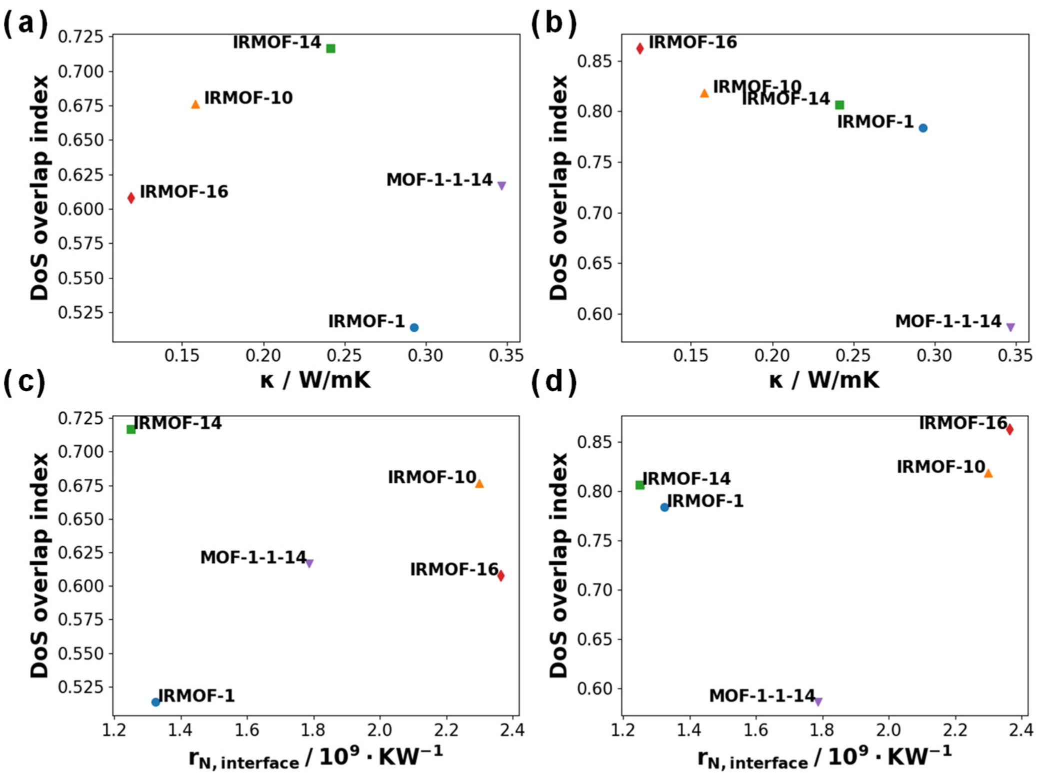

Figure S19. Overlap indices of the projected densities of states between linker and node for the investigated MOFs as a function of the thermal conductivity (a and b) and as a function of the interface transport-channel insulance (c and d). In (a and c) only modes with frequencies up to 9 THz and in (b and d) only modes with frequencies up to 3 THz were included. For the case of MOF-1-1-14 only the overlap of the projected densities of states between the pyrene-based linker with the node was used for the evaluation.

S4.3 Phonon band structures

The phonons of the materials investigated here determine heat transport properties. Even though the thermal conductivity is an inherently anharmonic property, harmonic phonon band structures still contain information on two of the major quantities determining the thermal conductivity: the phonon group velocities $\boldsymbol{v}_{g,i}$ and the mode heat capacities C_i. This becomes apparent from the Peierls-Boltzmann transport equation under relaxation time approximation for heat transport[36]:

$$\boldsymbol{\kappa} = \frac{1}{NV}\sum_{\lambda} C_\lambda(T)\boldsymbol{v}_{g,\lambda} \otimes \boldsymbol{v}_{g,\lambda}\tau_\lambda \,. \tag{S22}$$

Here, λ is the mode index, N is the number of unit cell, V is the system volume and τ_i are the single mode phonon relaxation times. Even though the latter cannot be obtained from calculations based on the harmonic approximation, it is evident that the group velocities (which just arise from the slope of the phonon band structure) are crucial since they enter the equation squared. Additionally, the mode heat capacities emerge from the density of states. These properties can be qualitatively estimated by looking at the phonon band structures, which are shown for the investigated systems – including the total density of states, which is the sum of the projected densities of states from section S4.2 – in Figure S20, Figure S21 and Figure S22. They were evaluated on 2×2×2 supercells using the fitted force field potentials. Based on these band structures it becomes evident that the acoustic modes are most likely those that carry the majority of heat for these systems. For all systems, there is also a large quantity of very flat phonon modes in the low frequency region intersecting with the acoustic phonons. Many of these modes correspond to rotational motions of the linkers, which shift to lower frequencies and increase in number for longer linkers. This is what makes it difficult to observe clear trends in the phonon density

of states, as discussed in the previous section. Aside of the true crossing of the acoustic modes, also some avoided crossings can be observed for all the systems. For example, at 1.0-1.5 THz for IRMOF-1. This is also an aspect that normally leads to a reduction in the thermal conductivity [37,38] and that makes it more difficult to analyze the individual acoustic modes. It should be noted, that a more thorough analysis of phonon band structures for similar Mg-based systems is provided in [8].

Figure S23 shows frequency dependent contributions of the group velocities and the mode heat capacities to the thermal conductivity for the individual systems. $C_\lambda(T)$ was calculated with the following expression:

$$C_\lambda(T) = k_B \left(\frac{\hbar\omega_\lambda}{k_B T}\right)^2 \cdot \frac{e^{\frac{\hbar\omega_\lambda}{k_B T}}}{\left(e^{\frac{\hbar\omega_\lambda}{k_B T}} - 1\right)^2} \tag{S23}$$

Here, ω_λ is the frequency of phonon mode λ. The total sums of the group velocities behave very similarly as the total thermal conductivities obtained from NEMD simulations. However, IRMOF-10 and IRMOF-16 have a very similar group velocity sum despite showing different thermal conductivities. This does not necessarily indicate a difference in phonon lifetimes, as those tend to be lower at higher frequencies and up to 17 THz there is also a visible difference in the group velocity sums of IRMOF-10 and IRMOF-16. Generally, it is very difficult to analyze the contributions of certain phonon modes or certain types of phonon modes, unless one has the full picture including the phonon relaxation times. This, however, exceeds the scope of this work.

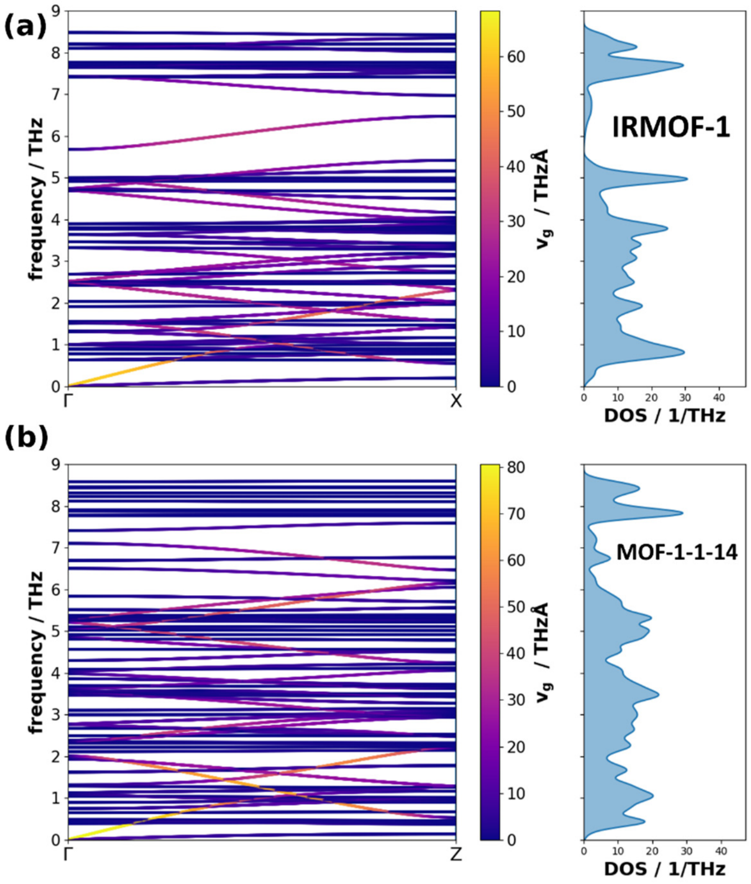

Figure S20. Low frequency phonon band structures of IRMOF-1 (a) and MOF-1-1-14 (in direction of the pyrene linkers Z) (b). The band structures were obtained for 2×2×2 primitive supercells. The color in the left panels shows the norm of the phonon group velocities at the respective wave vector.

In the right panel the phonon density of states evaluated on a 10×10×10 mesh is shown for the same frequency region.

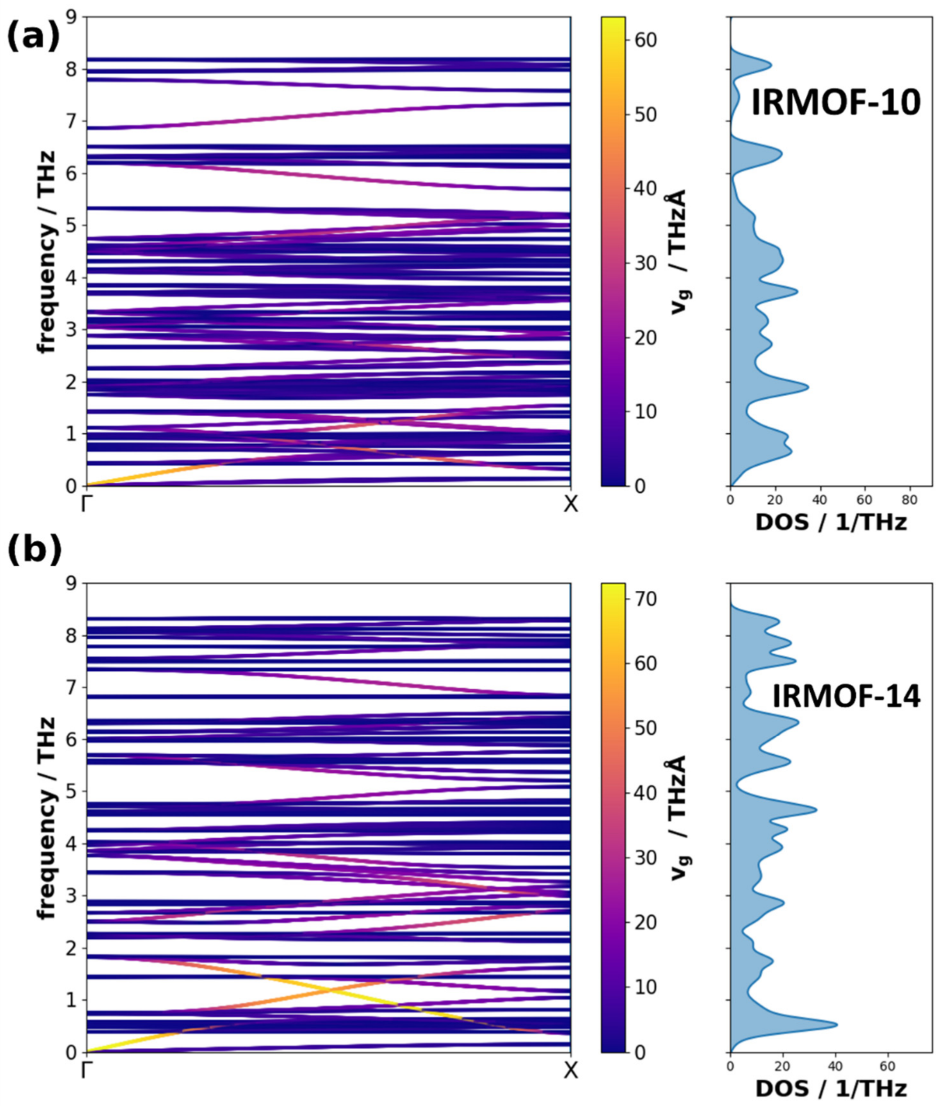

Figure S21. Low frequency phonon band structures of IRMOF-10 (a) and IRMOF-14 (b). The band structures were obtained for 2×2×2 primitive supercells. The color in the left panels shows the norm of the phonon group velocities at the respective wave vector. In the right panel the phonon density of states evaluated on a 10×10×10 mesh is shown for the same frequency region.

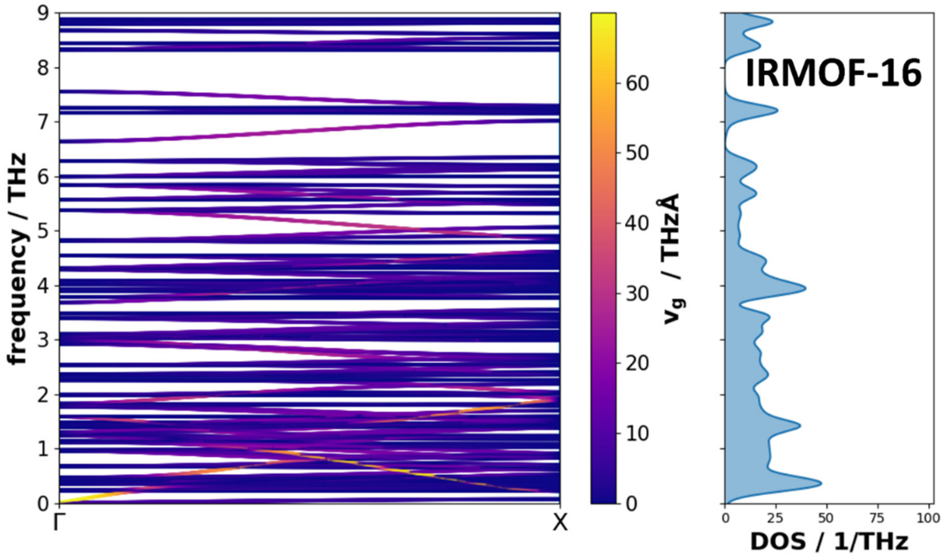

Figure S22. Low frequency phonon band structures of IRMOF-16. The band structure was obtained for a 2×2×2 primitive supercell. The color in the left panels shows the norm of the phonon group velocities at the respective wave vector. In the right panel the phonon density of states evaluated on a 10×10×10 mesh is shown for the same frequency region.

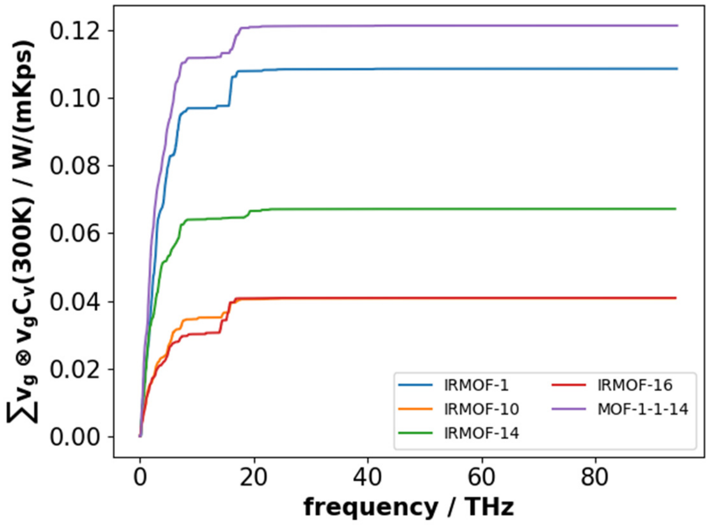

Figure S23. Contributions of the group velocities and mode heat capacities to the total thermal conductivity based on the Peierls-Boltzmann transport equation in the relaxation time approximation for including phonons up to a given frequency (the plotted quantity is proportional to a summation of thermal conductivity contributions up to a given frequency while at the same time setting all relaxation times to the same value). The values were evaluated on a 10×10×10 mesh of q-points.

S5. Setup and evaluation of the model systems

In order to compute linker length dependent data without the interreference of changes in the material's "chemical details", we used simple, yet instructive model systems. They are, in fact, similar to the one used by Babaei et al. [39] with a different layout of the inorganic node that we found to more accurately describe the situation at the node-linker interface.

S5.1 Setup of the model systems

The goal of the model systems is to provide an environment that allows a straightforward length expansion of the linkers by changing as little as possible of the other aspects of the system. The system used by Babaei et al. [39] simply consisted of straight,

linearly connected linkers that were connected to each other in a 90° angle by individual "virtual node atoms". Such a model system is shown in Figure S24a while Figure S24b shows the local effective temperature profile from a NEMD simulation performed for a corresponding 16×4×4 supercell at 300 K with 11 virtual atoms in the linear linker. In the temperature profile a temperature step can be seen similar to what occurs for the real MOFs between node and linker. And if we use this model system and extend the linker in only heat transport direction or in all directions and evaluate the thermal resistance contributions, a similar effect can be observed as for the real MOFs. In Figure S25 we can see an increasing contribution of the thermal interface resistance for longer linker lengths. Note, that deviating from the other thermal resistance contributions reported in the main manuscript of this work, the values reported in Figure S23 were not corrected for finite size effects and were only obtained for supercells of similar length. This is also the primary reason why the errors are so much smaller for these systems than for those shown in the main manuscript. However, there is a major difference to the real MOFs: there is no node contribution since the "node" is only a single virtual atom thick. This is why we found this type of model system to be suboptimal for the primary comparison. In the following, we will discuss the general functional definition of the terms in the model systems, compare a few candidates with slightly different "model-nodes" (to achieve a larger spatial extend of the node which leads to temperature profiles more similar to the real MOFs; see Figure 6 in the main paper), and we will also provide our reasoning for our choice of the type of model systems for the analysis.

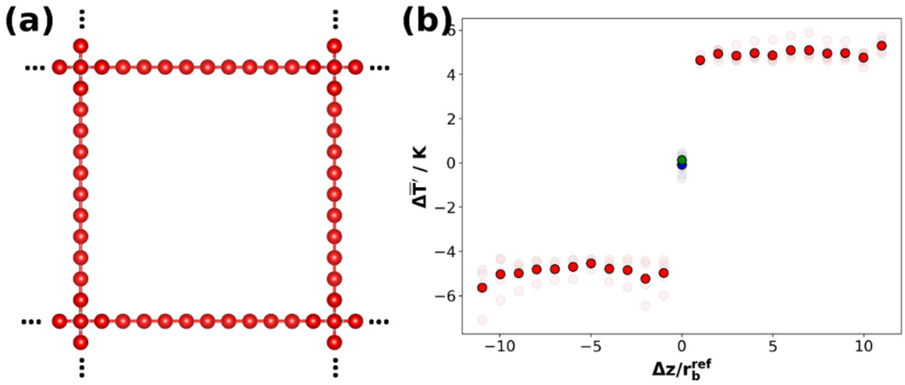

Figure S24. Structure of a model system with orthogonal connections of the linkers (a) and the corresponding averaged local effective temperature profile obtained from a NEMD simulation for a 16×4×4 supercell of a model system with 11 virtual linker atoms at 300 K (b). The different colors of indicate the virtual atoms of the linker in heat transport direction (red), virtual atoms of the linkers perpendicular to the heat transport direction (blue) and virtual node atoms (green). The transparent atoms in the background represent the individual atomic local effective temperatures before averaging over equivalent segments. The length values Δz in (b) were normalized by the bond length r_b^{ref} in the model systems.

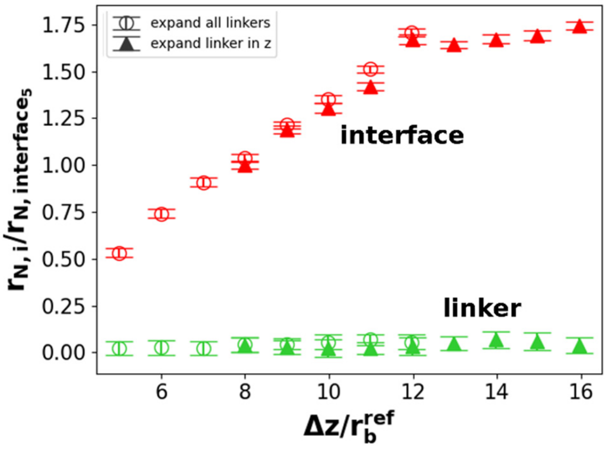

Figure S25. Linker length dependent relative thermal transport-channel insulance contributions for the interface and linkers for the simple model system with linkers extended only in heat transport direction (open symbols) and for linkers expanded in all directions (filled symbols). The interface transport-channel insulance for the model systems with 5 "virtual atoms" in the linker (corresponding to Dz= 7.416 r_b^{ref}) was used for the normalization of the y-axis. The supercells used in the NEMD simulations were chosen to result in simulation boxes of similar lengths and were in ascending order of linker length: 26×4×4, 21×4×4, 18×4×4, 16×4×4, 14×4×4, 13×4×4, 12×4×4, 11×4×4, 10×4×4, 9×4×4, 9×4×4, 8×4×4. The error bars result from the standard error of the linear fit of the thermal linker transport-channel insulance (note, that this choice of error bars is different than for the finite size corrected values used throughout this work, where the standard error of the extrapolation to the infinite size limit was used). The length values Δz were normalized by the bond length r_b^{ref} in the model systems.

The interatomic potential for the model systems shown here only consists of the bond stretching and angle bending parameter terms listed in section S2.1. The parameters were the same for all atoms in the model and were chosen to be similar to the parameters for real MOFs (actual properties will naturally differ as a MOF has different interaction strengths within a linker than within a node):

- The reference bond length r_b^{ref} amounts to 1.43 Å
- The bond term constant k_b amounts to 3 mdyn/Å
- The reference bending angle is chosen to maintain the general equilibrium structure of the system, i.e., to maintain 90° angles between the linkers or 45° between the linkers and the edges of the node octahedron.
- The angle term constant k_a is chosen to be 0.5 mdyn/(Å rad)

In the course of our simulations, we tested several different types of model systems differing in particular in the structures of the nodes. Two of these model systems are shown in Figure S26. The upper model system replaces the singular virtual node atom with a node octahedron in which each virtual node atom is connected to four other virtual node atoms and to one virtual linker atom. The other model system in Figure S26b contains an additional virtual atom in the center of the node, which is bonded to all six other virtual atoms in the node. In Figure S27b a more complex model system with an additional virtual atom between the bonded virtual atoms of the nodes is shown. These new virtual atoms are supposed to emulate the role of the Zn atoms in the real MOFs. In a final test, an even more complex model system was studied that included an additional central virtual atom (representing the inner oxygen atom in the real MOF), which is bonded to all the 8 nearest virtual node atoms (see Figure S28b).

For all of these model systems, the differences in phonon band structures were analyzed to estimate the importance of the structural modifications for heat transport. Figure S26c displays the phonon band structures in the direction along the linker for the model systems based on simple octahedral nodes with and without the central atom. It can be seen that some of the modes are shifted slightly between the two structures in terms of

frequency, but overall, the bands are very similar. Especially the acoustic bands, which are typically the modes most important for heat transport (due to their high group velocities), are almost identical. Figure S27c shows the phonon band structure comparison of the normal model system (the one from Figure S27a, which was also used in the main paper) and a more complex model system with larger nodes. Again, the phonon bands are relatively similar for both systems. The frequencies of some of the optical modes are shifted, but the general shapes of the bands prevail. Also, the slopes of the bands of the important acoustic modes are very similar. Additionally, the comparison of the band structures between the complex model system with and without a central atom contained in Figure S28c shows that the phonon bands hardly differ in the low frequency region.

Since we do not observe any fundamental differences in the phonon band structures between the model systems with varying degrees of complexity, for the further investigations we opted for using simplest system which produces temperature profiles similar to the real MOFs. This is the system shown in Figure S26a.

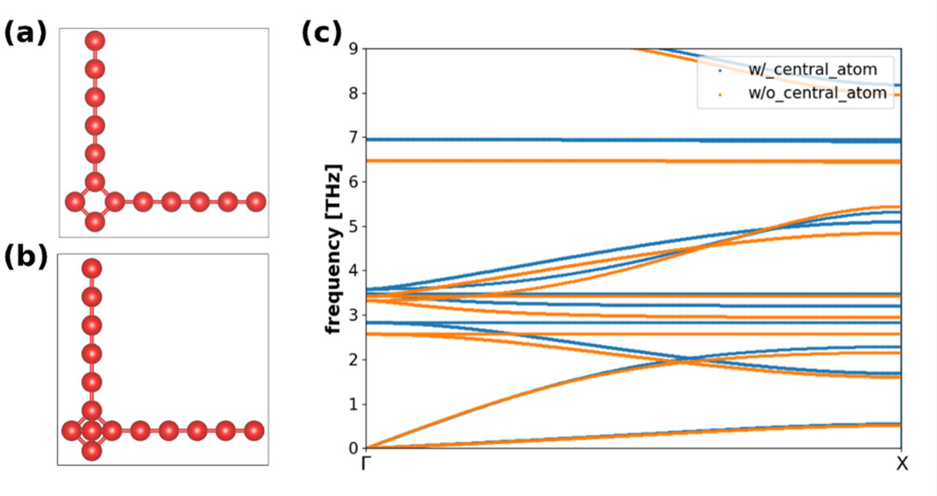

Figure S26. Cross-section of the model system used in the main paper (a) and a model system with a central atom in the node (b). The linkers perpendicular to the cross-section and the virtual atoms completing the octahedral shape of the nodes are not shown for the sake of clarity. In (c) the low frequency phonon band structures in X direction (the direction parallel to a linker) are compared for the two systems.

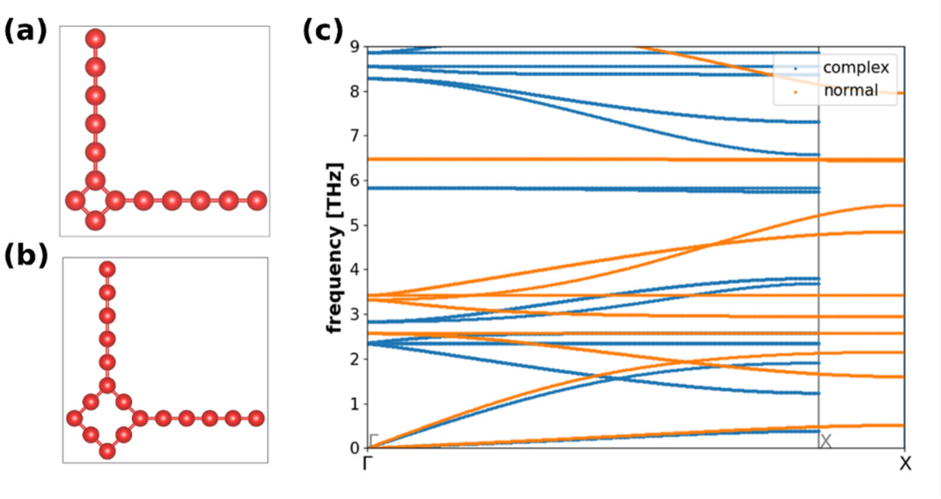

Figure S27. Cross-section of the model system used in the main paper (a) and with additional virtual atoms in the node (b). The linkers perpendicular to the cross-section and the virtual atoms completing the octahedral shape of the nodes are not shown for the sake of clarity. In (c) the low frequency phonon band structures in X direction (the direction parallel to a linker) are compared for the two systems. The different sizes of the unit cells of the model systems lead to a different extents of the Brillouin zone.

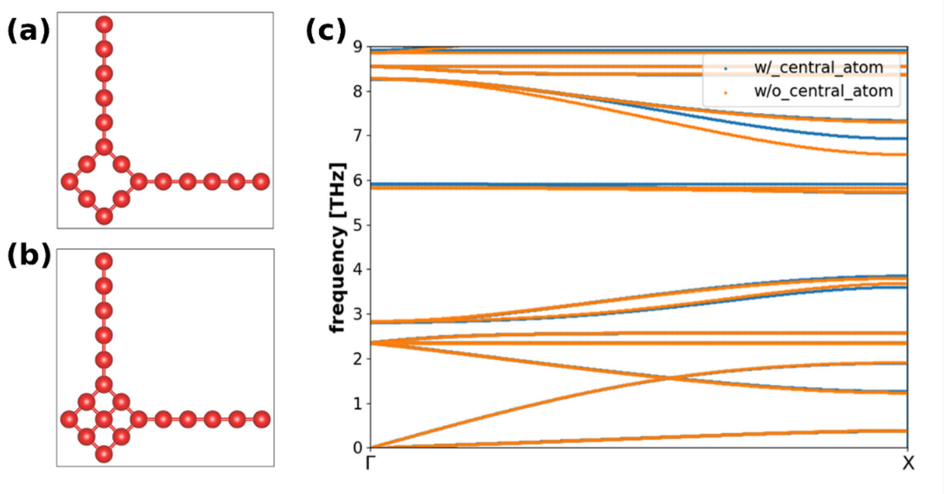

Figure S28. Cross-section of a model system with an octahedral node and an increased number of virtual atoms without (a) and with central virtual atom in the node (b). The linkers perpendicular to the cross-section and the virtual atoms completing the octahedral shape of the nodes are not shown for the sake of clarity. In (c) the low frequency phonon band structures in X direction (the direction parallel to a linker) are compared for the two model systems.

S5.2 NEMD simulations

In the following, data on the NEMD simulations on the model systems beyond those already shown in the main manuscript will be provided. The settings used for the simulations are mostly analogous to those for the actual MOFs described in section S3.2. The thermostats used for the models, however, spanned one unit cell of the model system each (which is equivalent to the real systems in terms of the number of nodes and linkers contained in the thermostat region) bearing in mind that the conventional unit cells of the studied MOFs comprise two linkers in each Cartesian direction (see discussion in section 3.3 in the main paper). Due to the periodicity and differences in the cell lengths of the systems, it is not possible to achieve perfectly equal cell length for the visualization of the local effective temperature profiles. Table S6 shows the actual supercells and the total cell lengths used for the simulations. For the systems in which the linker is only extended in one direction, cross-sectional linkers consisting of 5 atoms were used. For the two smallest systems with linker lengths changed in all directions thicker super cells perpendicular to heat transport direction were used to achieve convergence. In Figure S29 the local effective temperature profiles for the model systems with linkers expanded only in heat transport direction (which were not included in the main paper) can be seen.

Table S6. List of supercells used for the model systems to visualize the temperature profiles using NEMD simulations. For the two smallest systems, larger supercells were required perpendicular to heat transport direction. The length values in this table are given in Å (based on our chosen value for the bond length of 1.43 Å) to be more comparable to the actual MOFs.

Number of atoms in the linker	Supercell used	Length / Å
2	52×4×4, 52×8×8	328.7
3	42×4×4, 42×6×6	325.5
4	36×4×4	330.4
5	32×4×4	339.3
6	28×4×4	336.9
7	26×4×4	349.9
8	24×4×4	357.1
9	22×4×4	358.7

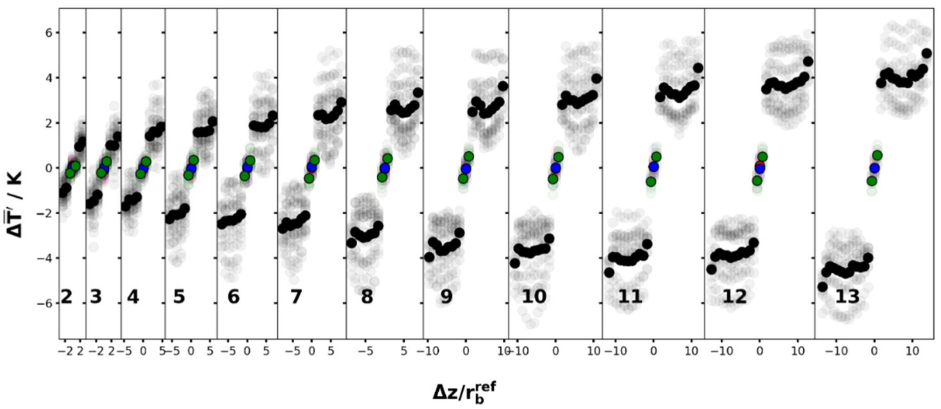

Figure S29. Local effective temperature profiles of a node and its adjacent linkers obtained from NEMD simulations for the model systems with linkers systematically extended in heat transport direction only. The temperature profiles have been averaged across all building blocks that are not within a distance of 50 Å from the center of the thermostat. The transparent data points in the background represent the local effective temperatures before averaging. The labels indicate the number of atoms in each linker. The total length of the simulation cells was fixed to be approximately equal for all systems, leading to the following super cells of the conventional unit cell: 2: 52×4×4, 3: 42×4×4, 4: 36×4×4, 5: 32×4×4, 6: 28×4×4, 7: 26×4×4, 8: 24×4×4, 9: 22×4×4. The colored symbols differentiate between atoms in the linkers in heat transport direction (black), atoms in the linkers perpendicular to heat transport direction (blue) and atoms comprising the node (green). The length values Δz were normalized by the bond length r_b^{ref} in the model systems.

Furthermore, the thermal conductivity values were corrected for finite size effects using 5 supercells of different lengths similarly to the actual MOFs (for details regarding the procedure see sectionS3.3). The fits used for the infinite size extrapolation of the model systems are shown in Figure S30 for different linkers extended in all directions and in Figure S31 for model systems with linker extended in heat transport direction only.

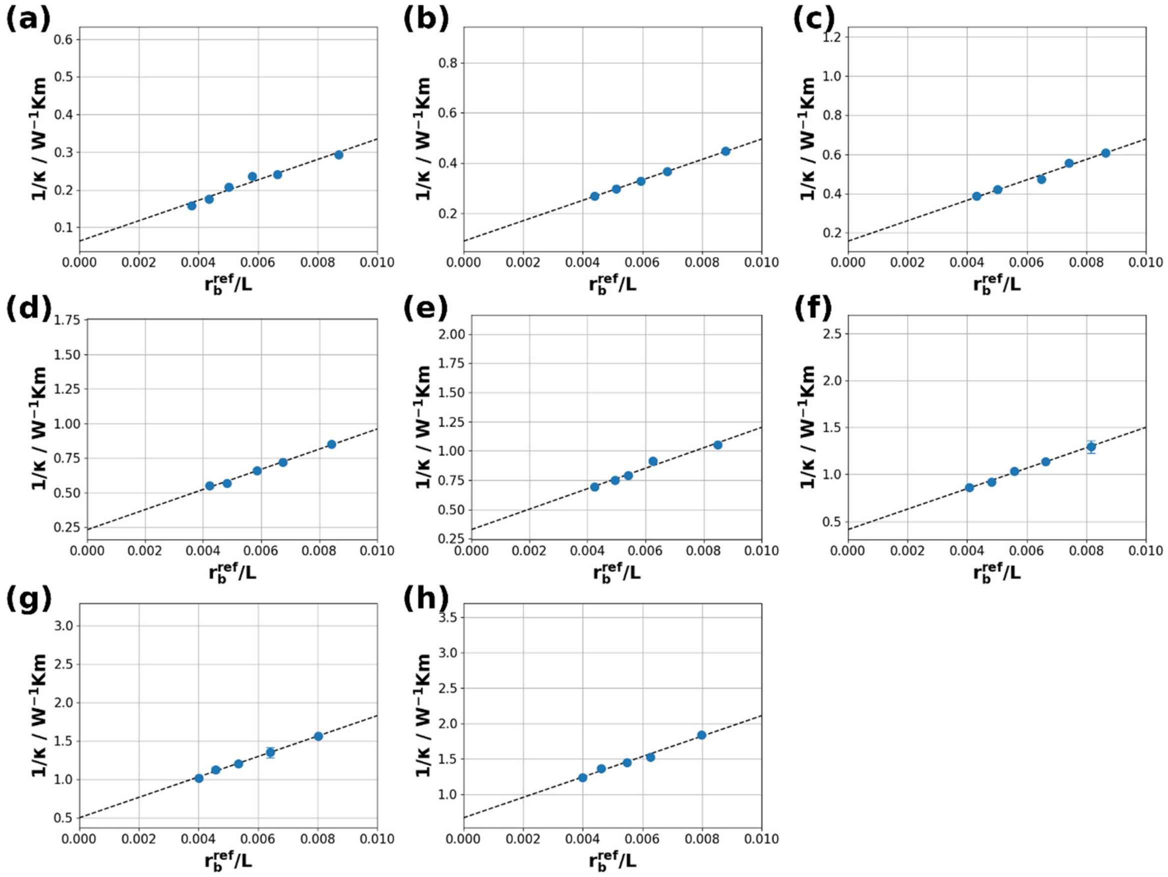

Figure S30. Inverse thermal conductivities as a function of inverse cell lengths relative to the model system reference bond length r_b^{ref} (blue) from NEMD simulations for the investigated model systems with linkers extended in all directions. The number of atoms in the linker for the different model systems shown here are (a) 2, (b) 3, (c) 4, (d) 5, (e) 6, (f) 7, (g) 8, and (h) 9. The dashed lines indicate the linear fits through the datapoints. The finite size corrected thermal conductivities can be extracted from the r_b^{ref}/L=0 intercept.

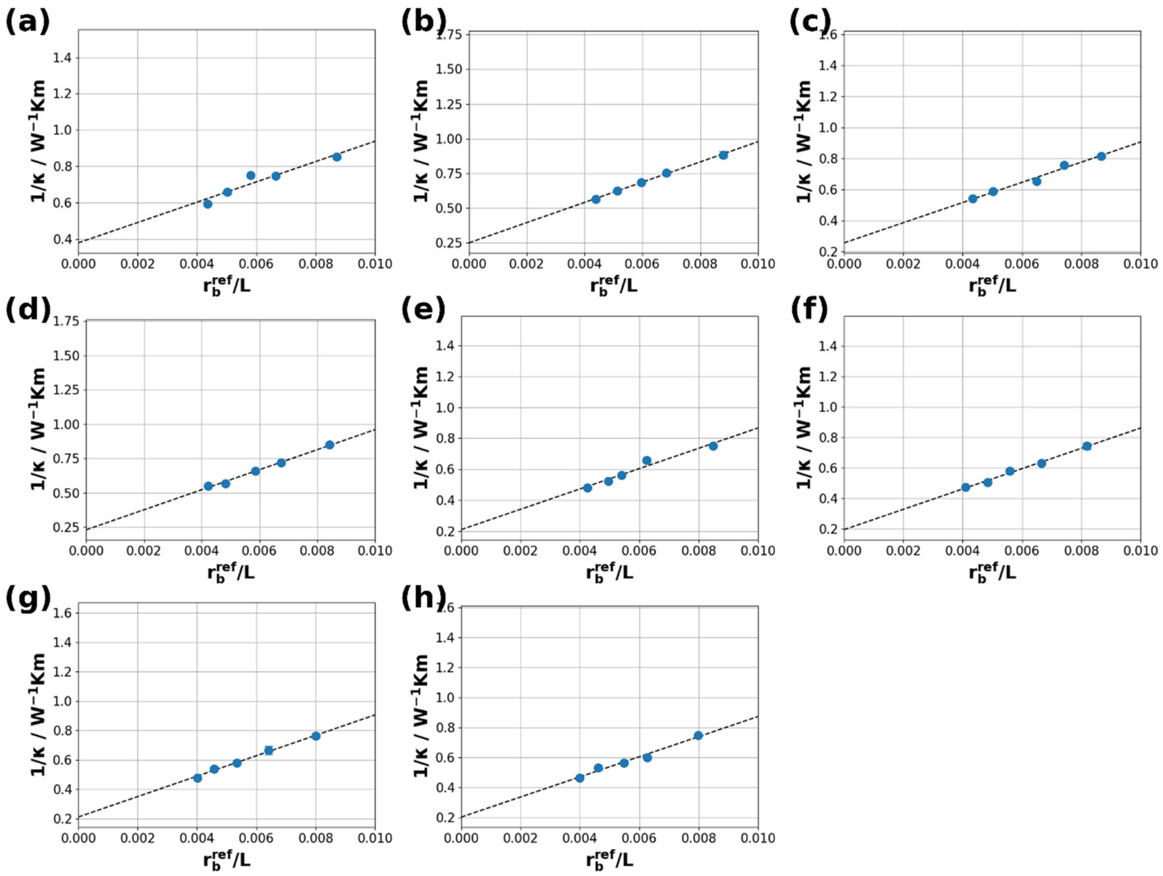

Figure S31. Inverse thermal conductivities as a function of inverse cell lengths relative to the model system reference bond length r_b^{ref} (blue) from NEMD simulations for the investigated model systems with linkers extended in heat transport direction only. The number of atoms in the linker for the different model systems shown here are (a) 2, (b) 3, (c) 4, (d) 5, (e) 6, (f) 7, (g) 8, and (h) 9. The number of atoms of the linkers in the cross-section perpendicular to the heat flux is 5. The dashed lines indicate the linear fits through the datapoints. The finite size corrected thermal conductivities can be extracted from the r_b^{ref}/L=0 intercept.

In the main manuscript, the thermal conductivity and transport channel insulance contributions are given relative to the values for the model system with 5 virtual atoms in each linker. For the sake of completeness, Figure S32 shows the absolute values obtained for the model system force fields as defined in section S5.1 and also as a function of the inverse cross section.

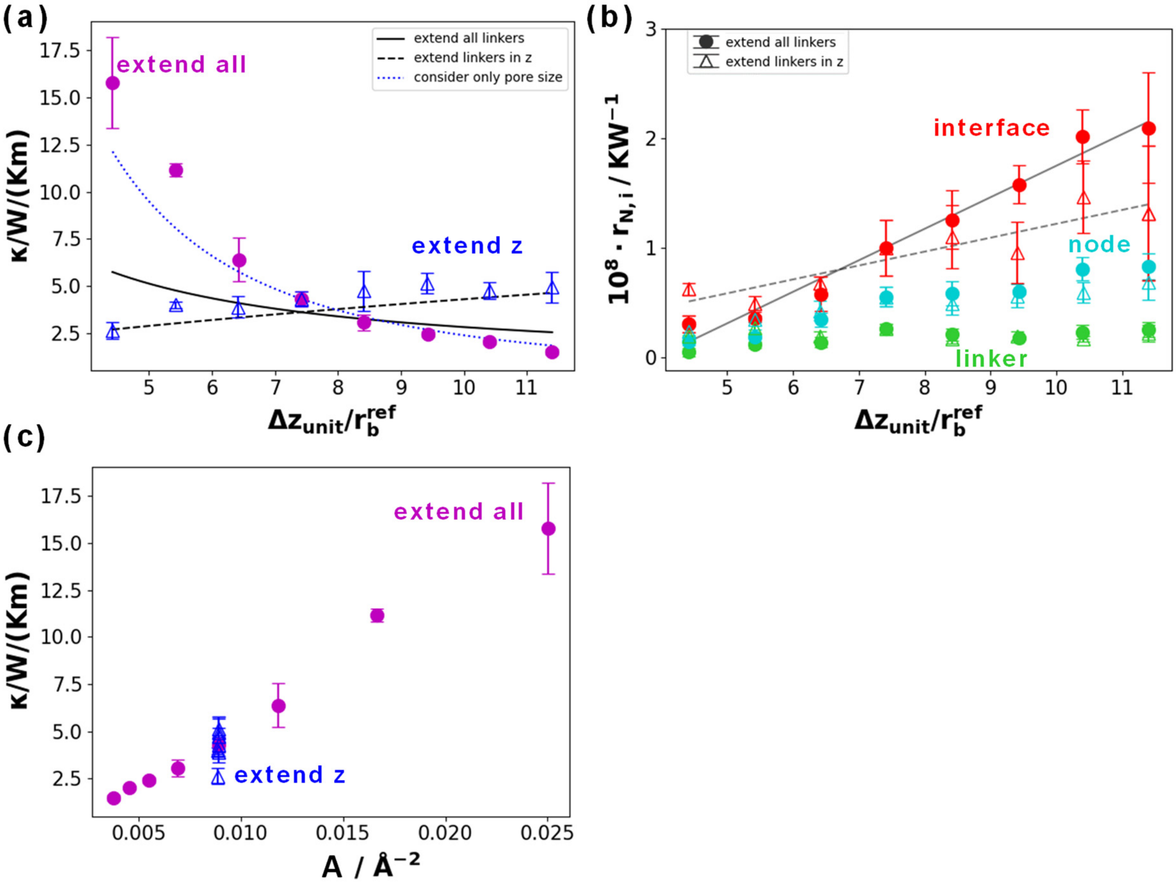

Figure S32. (a) NEMD-calculated thermal conductivities of the model systems with the numbers of "virtual atoms" varying in all directions (magenta) and varying only in one direction (blue). The error bars originate from the error of the infinite size extrapolation. The black lines indicate the predictions for extending linker lengths in all directions (solid line) and only in the heat transport direction (dashed line). The dotted blue line indicates the prediction when only considering the decrease in the linker density due to longer linkers. Panel (b) shows transport-channel-insulances of nodes, linkers, and interfaces of the model systems for extending the linkers in all directions (filled symbols) and for extending the linkers only in heat transport direction (empty symbols). The error bars originate from the standard error of the infinite size extrapolation. Linear fits through the interface transport-channel insulances are indicated in grey to provide a guide for the eye. The length values Δz in (a and b) are reported relative to the distance between "virtual atoms" in the model systems, r_b^{ref}. In (c) the thermal conductivity values for the model systems as a function of the inverse cross-sectional area of the "unit cell" of the model systems perpendicular to the heat-transport direction (A) is shown.

S5.3 Projected density of states

Figure S33 visualizes the projected density of states for the model systems with the linker extended in one direction and Figure S34 for the linker extended in all directions. The values are based on phonon calculations carried out by *phonopy* [14] and evaluated on a 10×10×10 mesh from force constants computed for a 2×2×2 super cell. The smearing parameter σ to approximate the delta distribution in the expression for the density of states (see equation S20) amounted to 0.1 THz.

In general, a similar trend can be observed as for the real systems. The longer linkers shift the primary peak towards lower frequencies. For the anisotropic systems the behavior is similar to MOF-1-1-14, where one can clearly see a separation of peaks associated with linkers of one or the other direction. A deviation from the situation in the actual MOFs is that the contributions of the nodes remain essentially the same for all the systems.

We attribute this to the fact that in the actual MOFs changing the linker also impacts the node-linker interaction, which is not the case for the model system.

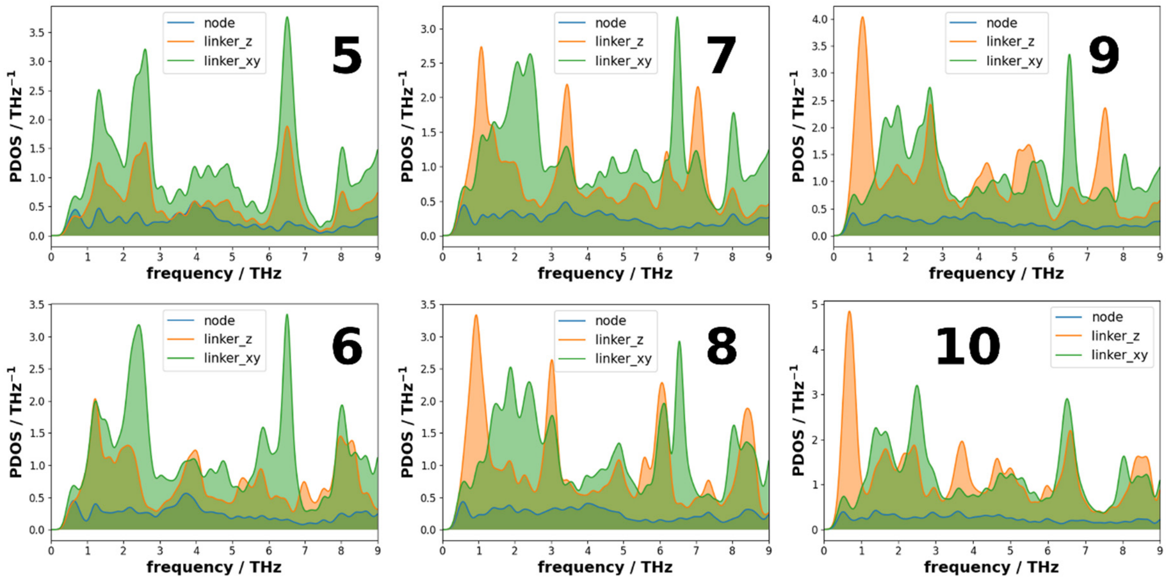

Figure S33. Projected density of states for the model systems and extending the linker in one direction. The black numbers indicate the number of atoms in the extended linker ("linker_z"). The linkers perpendicular to the heat-transport direction ("linker_xy") each contain 5 "virtual atoms".

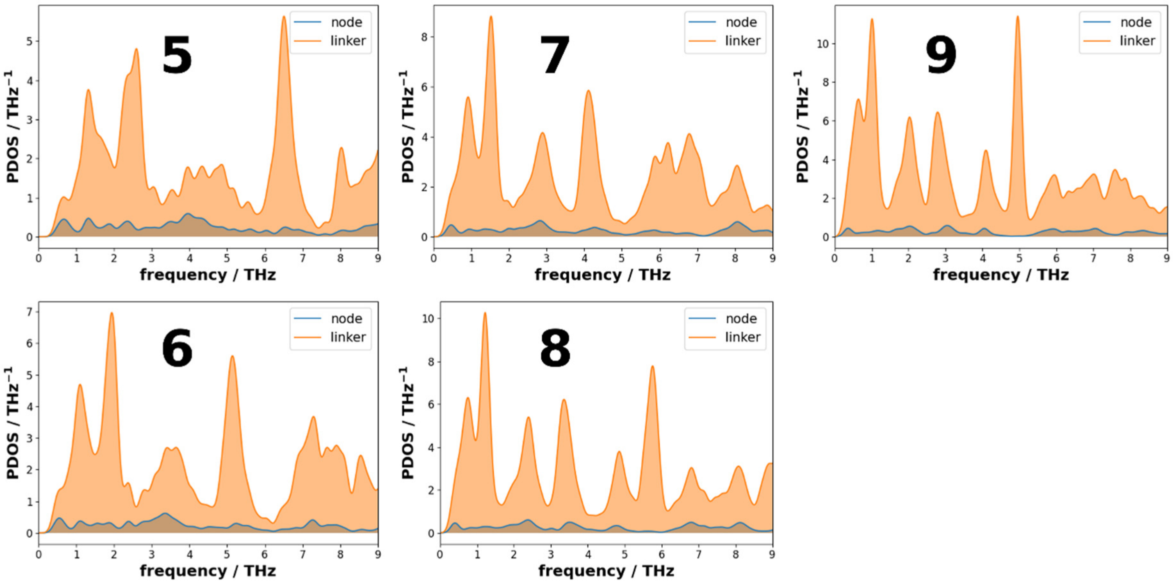

Figure S34. Projected density of states for the model systems and extending the linker in all directions. The black numbers indicate the number of atoms in each linker.

S5.4 Parameter scaling

To assess the impact of the choice of the model parameters on the thermal conductivity of the model system, they were systematically varied for a cubic model system with 9 atoms in each linker. The interaction parameters in the respective force fields were varied in the following way:

- the bond stretch interaction strength k_b^s (see equation S1) between "virtual atoms" in the linker was increased by a factor of two.

- the bond stretch interaction strength k_b^s (see equation S1) between "virtual atoms" in the node was increased by a factor of two
- the angle bending interaction strength k_a^b (see equation S2) between "virtual atoms" in the linker was increased by a factor of four
- the bond stretch interaction strength k_b^s (see equation S1) between "virtual atoms" in the linker only for linkers perpendicular to the heat transport direction was increased by a factor of two
- the angle bending interaction strength k_a^b (see equation S2) between "virtual atoms" in the linker only for linkers perpendicular to the heat transport direction was increased by a factor of four

The thermal conductivity results for a 11×4×4 supercell are shown in Table S7. The node bond strength and the linker angle strength have a significant impact, while changing the interaction strengths in the cross-sectional linkers is less relevant. This supports the rather natural assumption that the interactions in heat transport direction are far more important. However, all the thermal conductivity values are still within the same order of magnitude. Interestingly, the temperature profiles in the NEMD runs, especially in the nodes, are strongly affected by the bond and angle interaction strengths in the linker (see Figure S35). For these modifications in bond and angle interaction strengths, the temperature profile loses its step-like structure and becomes more of a periodic sequence of steeper or less steep slopes, which is a departure from the temperature profiles found in the actual MOFs. Therefore, we did not use these interaction strengths for the model systems in our analysis.

Table S7. Thermal conductivities for model systems with 9 atoms in the linker calculated for a 11×4×4 supercell with variations of specific force field interaction parameters.

System description	Thermal conductivity / W/mK
unmodified	0.81
linker angle bending interaction strength x4	1.47
linker bond stretch interaction strength x2	1.09
node bond stretch interaction strength x2	1.59
cross-sectional linker angle bending interaction strength x4	0.99
cross-sectional linker bond stretch interaction strength x2	0.82

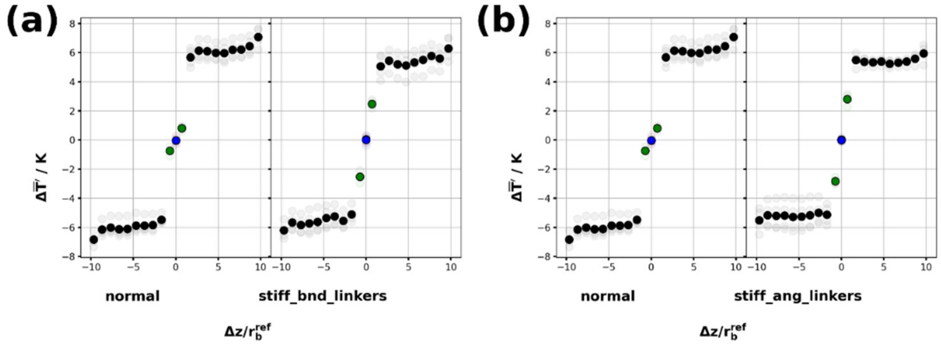

Figure S35. Local effective temperature profiles of a node and its adjacent linkers obtained from NEMD simulations for 11x4x4 super cells of model systems with 9 atoms in the linker with increased interaction strengths in the linker for the bond stretches (a) and for the angles (b). The temperature profiles were averaged across all building blocks that are not within a distance of 50 Å from the center of the thermostat. The transparent data points in the background represent the local effective temperatures before averaging. The labels indicate the number of atoms in each linker. The colored symbols differentiate between atoms in the linkers in heat transport direction (black), atoms in the linkers perpendicular to heat transport direction (blue) and atoms comprising the node (green). The length values Δz in (a,b) were normalized by the bond length r_b^{ref} in the model systems.

References

1. Kresse, G.; Hafner, J. Ab initio molecular dynamics for liquid metals. *Phys. Rev. B* **1993**, *47*, 558–561, doi:10.1103/PhysRevB.47.558.
2. Kresse, G.; Hafner, J. Ab initio molecular-dynamics simulation of the liquid-metalamorphous- semiconductor transition in germanium. *Phys. Rev. B* **1994**, *49*, 14251–14269, doi:10.1103/PhysRevB.49.14251.
3. Kresse, G.; Furthmüller, J. Efficient iterative schemes for ab initio total-energy calculations using a plane-wave basis set. *Phys. Rev. B - Condens. Matter Mater. Phys.* **1996**, *54*, 11169–11186, doi:10.1103/PhysRevB.54.11169.
4. Kresse, G.; Furthmüller, J. Efficiency of ab-initio total energy calculations for metals and semiconductors using a plane-wave basis set. *Comput. Mater. Sci.* **1996**, doi:10.1016/0927-0256(96)00008-0.
5. Blöchl, P.E. Projector augmented-wave method. *Phys. Rev. B* **1994**, *50*, 17953–17979, doi:10.1103/PhysRevB.50.17953.
6. Kresse, G.; Joubert, D. From ultrasoft pseudopotentials to the projector augmented-wave method. *Phys. Rev. B - Condens. Matter Mater. Phys.* **1999**, *59*, 1758–1775, doi:10.1103/PhysRevB.59.1758.
7. Perdew, J.P.; Burke, K.; Ernzerhof, M. Generalized gradient approximation made simple. *Phys. Rev. Lett.* **1996**, *77*, 3865–3868, doi:10.1103/PhysRevLett.77.3865.
8. Kamencek, T.; Bedoya-Martínez, N.; Zojer, E. Understanding phonon properties in isoreticular metal-organic frameworks from first principles. *Phys. Rev. Mater.* **2019**, *3*, 1–16, doi:10.1103/PhysRevMaterials.3.116003.
9. Kamencek, T.; Wieser, S.; Kojima, H.; Bedoya-Martínez, N.; Dürholt, J.P.; Schmid, R.; Zojer, E. Evaluating Computational Shortcuts in Supercell-Based Phonon Calculations of Molecular Crystals: The Instructive Case of Naphthalene. *J. Chem. Theory Comput.* **2020**, doi:10.1021/acs.jctc.0c00119.
10. Kresse, G.; Marsman, M.; Furthmüller, J. VASP the Guide.
11. Grimme, S.; Antony, J.; Ehrlich, S.; Krieg, H. A consistent and accurate ab initio parametrization of density functional dispersion correction (DFT-D) for the 94 elements H-Pu. *J. Chem. Phys.* **2010**, *132*, doi:10.1063/1.3382344.
12. Grimme, S.; Ehrlich, S.; Goerigk, L. Effect of the damping function in dispersion corrected density functional theory. *J. Comput. Chem.* **2011**, *32*, 1456–1465, doi:10.1002/jcc.21759.
13. Campañá, C.; Mussard, B.; Woo, T.K. Electrostatic potential derived atomic charges for periodic systems using a modified error functional. *J. Chem. Theory Comput.* **2009**, *5*, 2866–2878, doi:10.1021/ct9003405.
14. Togo, A.; Tanaka, I. First principles phonon calculations in materials science. *Scr. Mater.* **2015**, *108*, 1–5, doi:10.1016/j.scriptamat.2015.07.021.
15. Ziman, J.M. *Electrons and Phonons: The Theory of Transport Phenomena in Solids*; Oxford Clarendon Press, 2001; ISBN 0198507798 9780198507796.
16. Dürholt, J.P.; Fraux, G.; Coudert, F.X.; Schmid, R. Ab Initio Derived Force Fields for Zeolitic Imidazolate Frameworks: MOF-FF for ZIFs. *J. Chem. Theory Comput.* **2019**, *15*, 2420–2432, doi:10.1021/acs.jctc.8b01041.
17. Bureekaew, S.; Amirjalayer, S.; Tafipolsky, M.; Spickermann, C.; Roy, T.K.; Schmid, R. MOF-FF - A flexible first-principles derived force field for metal-organic frameworks. *Phys. Status Solidi Basic Res.* **2013**, *250*, 1128–1141, doi:10.1002/pssb.201248460.
18. Allinger, N.L.; Yuh, Y.H.; Lii, J.H. Molecular Mechanics. The MM3 Force Field for Hydrocarbons. 1. *J. Am. Chem. Soc.* **1989**, *111*, 8551–8566, doi:10.1021/ja00205a001.
19. Hansen, N. The CMA evolution strategy: A comparing review. *Stud. Fuzziness Soft Comput.* **2006**, *192*, 75–102, doi:10.1007/11007937_4.
20. Sun, H. COMPASS: An ab Initio Force-Field Optimized for Condensed-Phase ApplicationsOverview with Details on Alkane and Benzene Compounds. *J. Phys. Chem. B* **2002**, *102*, 7338–7364, doi:10.1021/jp980939v.
21. Rimmer, L.H.N.; Dove, M.T.; Goodwin, A.L.; Palmer, D.C. Acoustic phonons and negative thermal expansion in MOF-5. *Phys. Chem. Chem. Phys.* **2014**, *16*, 21144–21152, doi:10.1039/c4cp01701c.
22. Hockney, R. *Computer simulation Using Particles*; CRC Press: New York, 1989;
23. Lock, N.; Wu, Y.; Christensen, M.; Cameron, L.J.; Peterson, V.K.; Bridgeman, A.J.; Kepert, C.J.; Iversen, B.B. Elucidating negative thermal expansion in MOF-5. *J. Phys. Chem. C* **2010**, *114*, 16181–16186, doi:10.1021/jp103212z.
24. Li, Z.; Xiong, S.; Sievers, C.; Hu, Y.; Fan, Z.; Wei, N.; Bao, H.; Chen, S.; Donadio, D.; Ala-Nissila, T. Influence of thermostatting on nonequilibrium molecular dynamics simulations of heat conduction in solids. *J. Chem. Phys.* **2019**, *151*, doi:10.1063/1.5132543.
25. Huang, B.L.; McGaughey, A.J.H.; Kaviany, M. Thermal conductivity of metal-organic framework 5 (MOF-5): Part I. Molecular dynamics simulations. *Int. J. Heat Mass Transf.* **2007**, *50*, 393–404, doi:10.1016/j.ijheatmasstransfer.2006.10.002.

26. Huang, B.L.; Ni, Z.; Millward, A.; McGaughey, A.J.H.; Uher, C.; Kaviany, M.; Yaghi, O. Thermal conductivity of a metal-organic framework (MOF-5): Part II. Measurement. *Int. J. Heat Mass Transf.* **2007**, *50*, 405–411, doi:10.1016/j.ijheatmasstransfer.2006.10.001.
27. Schelling, P.K.; Phillpot, S.R.; Keblinski, P. Comparison of atomic-level simulation methods for computing thermal conductivity. *Phys. Rev. B - Condens. Matter Mater. Phys.* **2002**, *65*, 1–12, doi:10.1103/PhysRevB.65.144306.
28. Wieser, S.; Kamencek, T.; Dürholt, J.P.; Schmid, R.; Bedoya-Martínez, N.; Zojer, E. Identifying the Bottleneck for Heat Transport in Metal–Organic Frameworks. *Adv. Theory Simulations* **2020**, *2000211*, doi:10.1002/adts.202000211.
29. Addicoat, M.A.; Vankova, N.; Akter, I.F.; Heine, T. Extension of the universal force field to metal-organic frameworks. *J. Chem. Theory Comput.* **2014**, *10*, 880–891, doi:10.1021/ct400952t.
30. Rappé, A.K.; Casewit, C.J.; Colwell, K.S.; Goddard, W.A.; Skiff, W.M. UFF, a Full Periodic Table Force Field for Molecular Mechanics and Molecular Dynamics Simulations. *J. Am. Chem. Soc.* **1992**, *114*, 10024–10035, doi:10.1021/ja00051a040.
31. Mayo, S.L.; Olafson, B.D.; Goddard, W.A. DREIDING: A generic force field for molecular simulations. *J. Phys. Chem.* **1990**, *94*, 8897–8909, doi:10.1021/j100389a010.
32. Boyd, P.G.; Moosavi, S.M.; Witman, M.; Smit, B. Force-Field Prediction of Materials Properties in Metal-Organic Frameworks. *J. Phys. Chem. Lett.* **2017**, *8*, 357–363, doi:10.1021/acs.jpclett.6b02532.
33. Ju, S.; Liang, X.; Wang, S. Investigation of interfacial thermal resistance of bi-layer nanofilms by nonequilibrium molecular dynamics. *J. Phys. D. Appl. Phys.* **2010**, *43*, doi:10.1088/0022-3727/43/8/085407.
34. Ran, X.; Guo, Y.; Hu, Z.; Wang, M. Interfacial phonon transport through Si/Ge multilayer film using Monte Carlo scheme with spectral transmissivity. *Front. Energy Res.* **2018**, *6*, 1–9, doi:10.3389/fenrg.2018.00028.
35. Lamaire, A.; Wieme, J.; Hoffman, A.E.J.; Van Speybroeck, V. Atomistic insight in the flexibility and heat transport properties of the stimuli-responsive metal-organic framework MIL-53(Al) for water-adsorption applications using molecular simulations. *Faraday Discuss.* **2020**, doi:10.1039/d0fd00025f.
36. Togo, A.; Chaput, L.; Tanaka, I. Distributions of phonon lifetimes in Brillouin zones. *Phys. Rev. B - Condens. Matter Mater. Phys.* **2015**, *91*, doi:10.1103/PhysRevB.91.094306.
37. Li, W.; Mingo, N. Ultralow lattice thermal conductivity of the fully filled skutterudite YbFe<inf>4</inf>Sb<inf>12</inf> due to the flat avoided-crossing filler modes. *Phys. Rev. B - Condens. Matter Mater. Phys.* **2015**, *91*, 1–6, doi:10.1103/PhysRevB.91.144304.
38. Li, W.; Carrete, J.; Madsen, G.K.H.; Mingo, N. Influence of the optical-acoustic phonon hybridization on phonon scattering and thermal conductivity. *Phys. Rev. B* **2016**, *93*, 1–5, doi:10.1103/PhysRevB.93.205203.
39. Babaei, H.; McGaughey, A.J.H.; Wilmer, C.E. Effect of pore size and shape on the thermal conductivity of metal-organic frameworks. *Chem. Sci.* **2016**, *8*, 583–589, doi:10.1039/C6SC03704F.
